# Clinical comparison of two surgical techniques in obtaining complete root coverage of single RT1 gingival recessions

**DOI:** 10.1007/s00784-025-06491-2

**Published:** 2025-09-10

**Authors:** Michele Paolantonio, Paolo De Ninis, Pasquale Santamaria, Giuseppe Balice, Matteo Serroni, Bruna Sinjari, Alessio Frisone, Stefania Di Gregorio, Luigi Romano, Giovanna Murmura, Beatrice Femminella

**Affiliations:** 1 Department of Innovative Technologies in Medicine & Dentistry, “G. D’Annunzio” University, Via Dei Vestini 31, Chieti, Italy; 2 ”Luisa D’Annunzio” Institute for High Culture, Pescara, Italy; 3 Periodontology Unit, Centre for Host Microbiome Interactions, Faculty of Dentistry, Oral & Craniofacial Sciences, King’s College London, London, UK; 4 Department of Periodontics and Preventive Dentistry, University of Pittsburgh School of Dental Medicine, Pittsburgh, PA USA

**Keywords:** Clinical Trial, Gingival Recession, Grafts, Gingival thickness, Plastic Periodontal Surgery

## Abstract

**Objectives:**

This study aimed to compare the efficacy of the full-thickness palatal graft technique (FTPGT) and the coronally advanced flap with subepithelial connective tissue graft (CAF + SCTG) in achieving complete root coverage (CRC) in single gingival recessions (GR).

**Methods:**

Forty healthy patients with a single RT1 GR were randomized into two groups: 20 treated with CAF + SCTG and 20 with FTPGT. Baseline and 12-month measurements of GR, keratinized tissue width (KTW), probing depth (PD), clinical attachment level (CAL), and gingival thickness (GT) were recorded. CRC percentage, root coverage percentage (RC%), Root Coverage Esthetic Score (RES), patient-reported outcomes (PROs), and palatal wound healing were evaluated.

**Results:**

At the 12-month evaluation, 19 patients in the FTPGT group achieved CRC compared to 12 in the CAF + SCTG group (*p* < 0.004). FTPGT showed significantly more GR reduction (0.7 mm ± 0.19), greater CAL gain (0.65 mm ± 0.20), increased GT (0.99 mm ± 0.27), *p* < 0.001, and KTW gain (2.95 mm ± 0.5), and a higher RC% (12.71 ± 3.82), *p* < 0.002. PROs did not significantly differ between treatments, nor did palatal healing parameters. CAF + SCTG showed superior RES scores compared to FTPGT (*p* < 0.0003).

**Conclusions:**

FTPGT is more effective than CAF + SCTG in achieving CRC and improving GT, KTW, CAL gain, and GR reduction, particularly in deep recessions. CAF + SCTG provides superior esthetic outcomes. PROs were comparable between the two techniques, but palatal healing was slower in the FTPGT group.

**Clinical relevance:**

FTPGT, especially in deep single recessions, could serve as an alternative to CAF + SCTG, as it is associated with greater CRC, greater GT and KTW. However, it is linked to slower healing of the palatal donor site.

Clincaltrial.gov registration NCT04028037.

**Supplementary Information:**

The online version contains supplementary material available at 10.1007/s00784-025-06491-2.

## Introduction

When the gingival margin is located apical to the cemento-enamel junction (CEJ), the clinician is faced with a gingival recession (GR) [1].

GRs occur as a result of bacterial plaque accumulation or traumatic action of oral hygiene instruments [2]. The latter factor is particularly observed in industrialized countries [3]; furthermore, untreated GRs have a high probability of progression over time [4].

The main consequences of GRs are potential esthetic discomfort, dental hypersensitivity (DH), oral hygiene difficulties, and root caries^2^. These conditions indicate the need for the surgical treatment of GRs [5].

The degree of success of surgical techniques to treat GRs is influenced by patient-related factors (i.e. smoking) and site-specific factors (i.e. recession depth and width, gingival phenotype) [2].

The ideal endpoint of periodontal plastic surgery is complete root coverage (CRC), as incomplete results may not resolve clinical problems (i.e. DH, esthetics) [6]. The new position of the gingival margin should make the CEJ not visible [7].

The wide variability of CRC in the literature results from heterogeneity in important variables influencing the clinical outcome, such as recession dimensions, surrounding tissue characteristics, patient smoking status, and surgical technique [6].

A recently proposed surgical technique using a very thick graft has achieved high percentages of CRC in single recessions: the full-thickness palatal graft technique (FTPGT) [8]. Therefore, the main outcome of this study was to compare the likelihood of obtaining CRC in single recessions using the FTPGT or the coronally advanced flap with subepithelial connective tissue graft (CAF + SCTG), currently considered the"gold standard"for root coverage [9].

## Materials and methods

### Study design and trial registration

This study presents the 12-month follow-up results of a randomized, controlled, parallel-arm, blinded clinical trial (ClinicalTrials.gov registration NCT04028037) on the efficacy of two surgical techniques for achieving CRC in RT1 GRs. Patients were divided into two groups: one treated with CAF + SCTG and the other with FTPGT. The study protocol was approved by the Ethical Committee of"G. D'Annunzio"University (No. 1075–28/10/2015), in accordance with the 1975 Declaration of Helsinki, as revised in Fortaleza in 2013.

The study began on November 19th, 2015, and final data from the last participants were collected in October 2018.

**Fig. 1:**
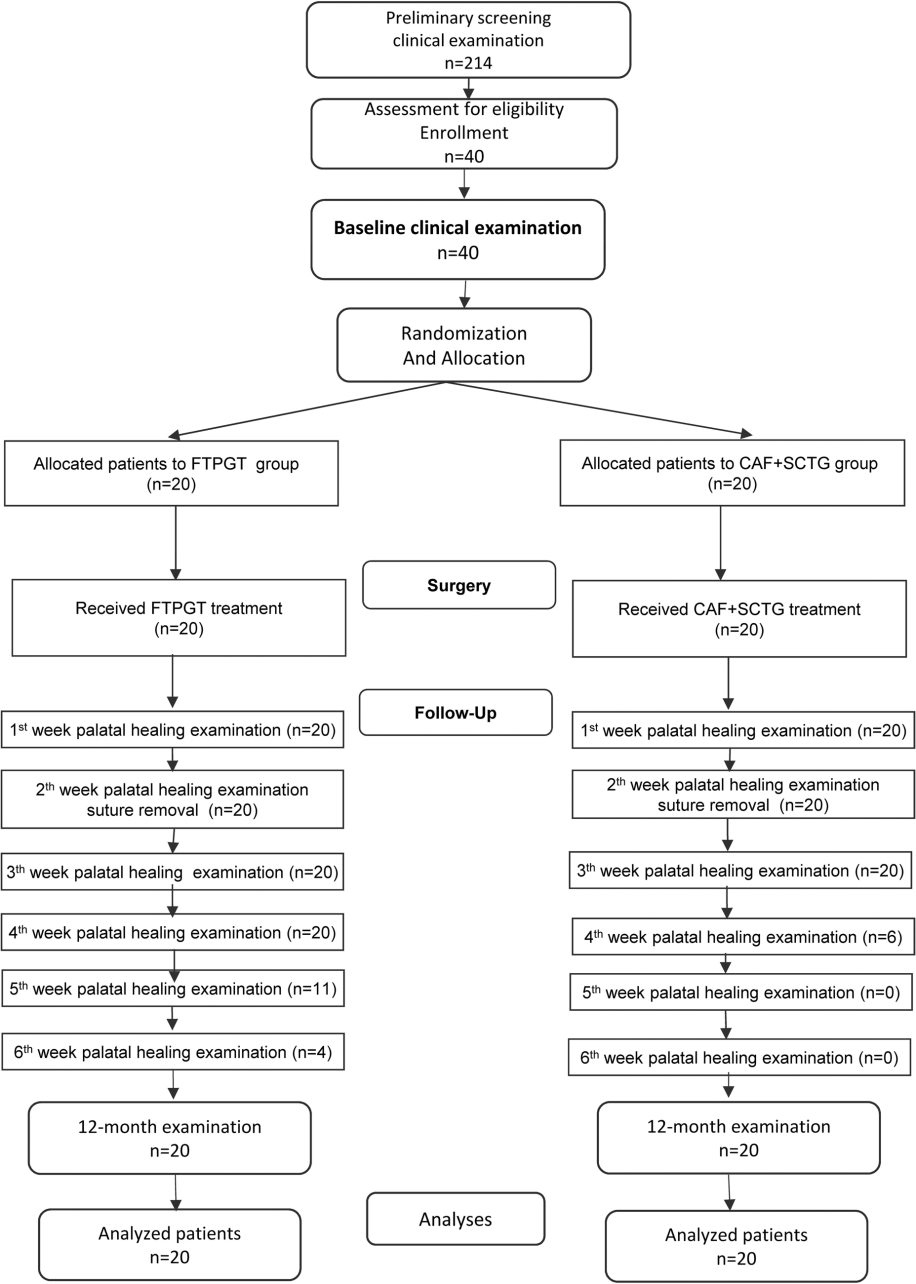
CONSORT diagram showing the study layout

Figure 1 shows the CONSORT flow diagram [10].

### Sample size

The sample size was calculated to compare two proportions: an expected CRC percentage of 95% and the 60% CRC percentage derived from our meta-analysis [Supplementary Material (SM) 1], based on the systematic review by Chambrone et al. regarding CAF + SCTG treatment at 12 months [6]. With α = 0.05 and β = 0.2, 18.42 patients (rounded up to 20) per group were needed (SM 2).

### Study population

Forty patients were selected from those diagnosed with at least one RT1 GR who presented at the Unit of Periodontology of the"G. D’Annunzio"University between October 2015 and July 2017.

Inclusion criteria: 1) systemically healthy; 2) no medications affecting periodontal status in the previous 6 months; 3) not pregnant or lactating; 4) never-smoker or former-smoker (≥ 10 years); 5) no previous periodontal plastic surgery; 6) full-mouth plaque score (FMPS) [11] and full-mouth bleeding score (FMBS) [12] < 15% at surgery; 7) at least 20 teeth without dental mobility; 8) no non-carious cervical or periapical lesions at experimental sites; 9) at least one RT1 buccal GR.

Each patient participated with a single recession.

After receiving comprehensive information, the patients signed a consent form.

### Randomization and blinding protocol

Random allocation was achieved using a computer-generated table. The study was single-blinded (patients) because the surgical technique could be identified by clinicians based on the postsurgical appearance.

### Clinical measurements

All clinical parameters were measured using the UNC-15 periodontal probe^1^ by the same investigator (LR) at baseline (T0) and 12 months later (T1). Sixty recessions were measured twice within 72 h; calibration was accepted when 90% of GR measurements were within 0.3 mm and 0.5 mm.

An occlusal acrylic stent was prepared for each patient, with a guiding groove made at the mid-buccal site of the experimental tooth. GR was measured from the CEJ to the gingival margin. Keratinized tissue width (KTW) was measured as the distance from the mid-buccal site of the gingival margin to the mucogingival junction (MGJ). Probing depth (PD) and clinical attachment level (CAL) were measured as the distance between the bottom of the sulcus and the gingival margin or the CEJ, respectively. All measurements were rounded to the nearest millimeter.

Gingival thickness (GT) was measured 1 mm apical to the sulcus depth, corresponding with the groove on the stent, using a #15 endodontic reamer^2^ inserted perpendicular to the gingiva until it reached the bone. A disk stop was placed in contact with the gingiva and fixed with cyanoacrylate. GT was the distance from the reamer’s tip to the silicon disk, measured with a digital caliper.^3^

The percentage of sites achieving CRC was recorded. CRC was assessed when the CEJ was not visible7. The percentage of root coverage (RC%) was calculated using the following formula: (〖GR〗_T0-〖GR〗_T1)/〖GR〗_T0.

### Patient-reported outcome measures

Patients were asked to record the number of painkiller tablets (400 mg ibuprofen**) taken during the first week.

At suture removal, patients reported the degree of overall discomfort (D) experienced on a VAS scale (0–10) and indicated the extent of changes in their feeding habits (CFH) caused by the palatal wound.

Dentine hypersensitivity (DH) was evaluated at T0 and T1 by the same examiner (LR) using the Schiff cold air sensitivity scale [13]. After protecting the adjacent teeth, a jet of air from a dental syringe at 60 psi from 10 mm was directed against the tooth’s cervical area. The patient’s response was then quantified on a 0–3 scale (“0” reaction to air stimulus; “1” reaction without requiring its termination; “2” reaction with a request to stop; “3” painful stimulus complaint).

Patient-reported esthetic satisfaction (PRES), quantified on a VAS scale (0–10), was recorded after comparing two standardized photographs showing the treated site plus two mesial and two distal teeth and the MGJ at T0 and T1. Overall treatment satisfaction (OTS) at T1 was assessed by asking each patient if, considering the result obtained and the pain experienced, they would undergo surgery again (yes/no).

### Esthetic outcome

The esthetic result was evaluated at the 12-month follow-up by the Root Coverage Esthetic Score (RES) [14].

### Palatal wound healing evaluation

The number of delayed bleeding episodes from the palatal wound (DWB) during the first postoperative week and, for each observation week, the time until complete re-epithelialization of the palatal wound (CWE) were recorded. CWE (yes/no) was evaluated by the peroxide test [15]. Re-epithelialization was considered complete if, after being sprinkled with 3% H_2_O_2_, no bubbles appeared.

### Pre-surgical treatment

Two months before surgery each patient underwent a professional mechanical plaque removal visit and individualized oral hygiene instructions.

### Surgical technique

**Fig. 2:**
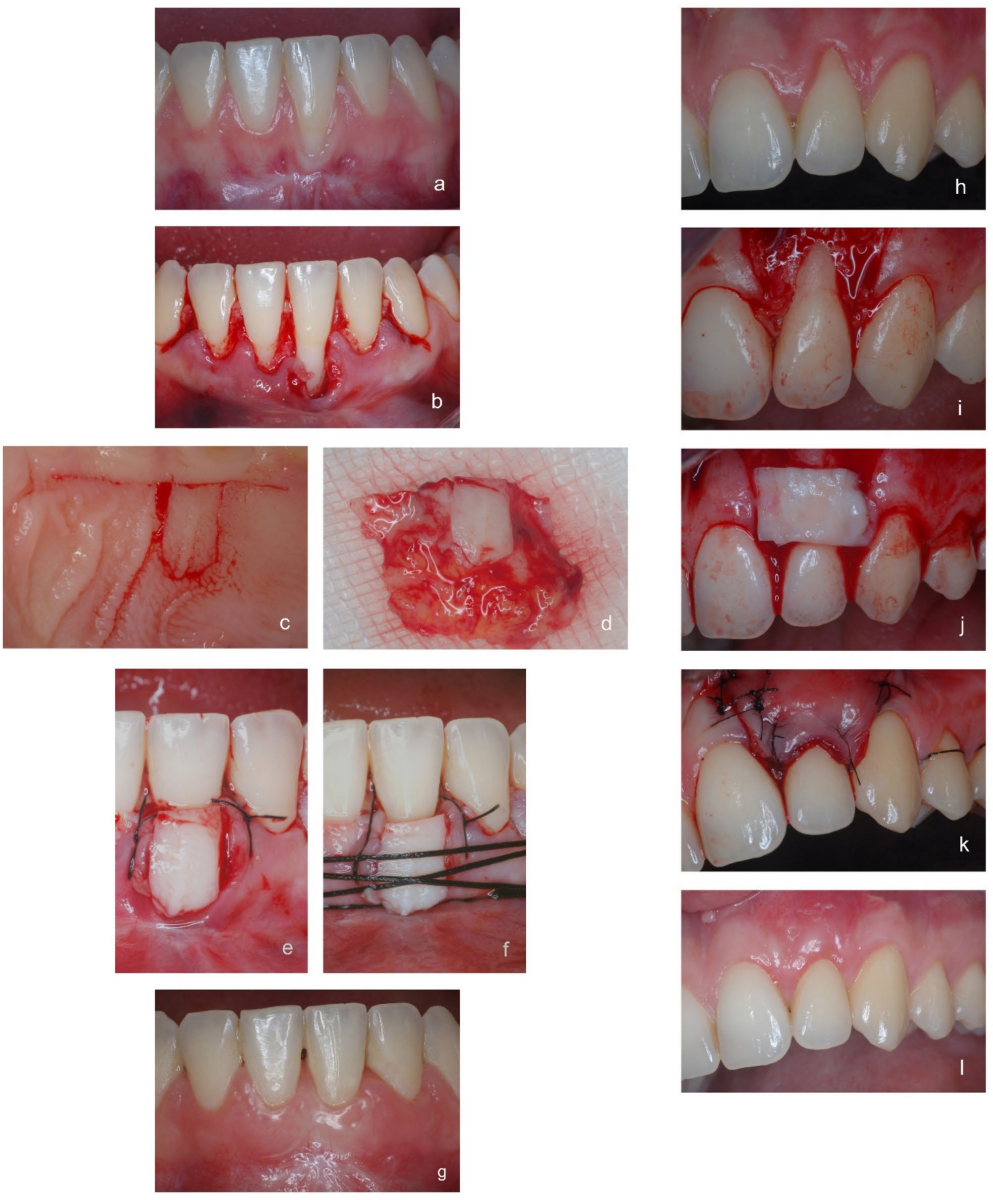
**a** FTPGT group. RT1 gingival recession at baseline; **b** FTPGT group. A split-thickness flap is raised from the gingival margin apically to the mucogingival junction; **c** FTPGT group. The palatal incision for graft harvesting; **d** FTPGT group. The palatal graft, including, in its central part, the entire thickness of the palatal soft tissues, from the epithelium to the periosteum; **e** FTPGT group. The graft is sutured under the gingival flap; **f**) FTPGT group. Compressive sutures stabilize the graft at the recipient site; **g** FTPGT group. The 12-month clinical result; **h** CAF + SCTG group. RT1 gingival recession at baseline; **i** CAF + SCTG group. A split-full-split thickness flap is raised; CAF + SCTG group. A connective tissue graft is placed on the exposed root; **k** CAF + SCTG group. The coronally advanced flap is sutured; **l** CAF + SCTG group. The 12-month clinical result

All surgical procedures were performed by the same experienced clinician (MP) and are shown in Fig. 2.

Root planing was accurately performed using sharp curettes after local anesthesia.

#### CAF + SCTG group

According to De Sanctis & Zucchelli, a tension-free trapezoidal flap was elevated by the split-full-split technique [16], and the anatomic papillae coronal to the horizontal incisions were de-epithelialized. A 1-mm thick connective tissue graft (SCTG) was harvested from the palate as an epithelialized graft and an haemostatic collagen sheet was placed and sutured onto the donor zone. The width of the graft was equal to the exposed root + 3 mm of connective tissue on each side. The height of the graft was equal to the distance between the buccal bone crest and the CEJ. After epithelium removal, the graft was positioned and sutured 1 mm apical to the cement–enamel junction with 5–0 resorbable sutures.^4^ The SCTG was then covered by the tension-free coronally positioned flap, sutured about 2 mm over the CEJ with 5–0 silk sutures^4^.

#### FTPGT group

The FTPGT was carried out according to Paolantonio et al. [8] At the recipient site an intrasulcular incision extending from the distal line-angle of the second tooth preceding the recession to the mesial line-angle of the second tooth following the recession, without vertical incisions, was made. A split-thickness flap, enclosing the interdental papillae, was raised from the gingival margin apically to the mucogingival junction, thus creating a well-vascularized envelope to receive the graft.

At the palatal donor site, a straight incision, 2-3 mm apical to the gingival margin and deep to the bone, was made from the distal line-angle of the cuspid to the mesial line-angle of the first molar. Starting and ending around the centre of this incision, a “U” shaped incision about 1–2 mm deep was made, with the convex side towards the palatine vault. The width of the"U"shaped incision was equal to the width of the GR while the length was about 1 mm greater. A split-thickness dissection of the area surrounding the “U” shaped incision was performed reaching the first incision coronally and extending 2–3 mm beyond the"U-shaped incision apically. This portion was then separated from the surrounding connective tissue by making 3 internal incisions (mesial, distal and medial) as in the Hurzeler and Weng technique [17]. The graft was removed by detaching it from the bony surface with a #1 Ochsenbein chisel. A haemostatic collagen sheet was inserted into the donor zone, and the wound margins were sutured along the primary incision with interrupted sutures. The graft consisted of an apico-lateral portion of connective tissue and periosteum, and of a central part representing the entire thickness of the palate.

This graft was inserted into the envelope preparation at the recipient site, and the interdental papillae were sutured back to their original position with a 5"0"silk suture.^5^ A compressive 2 “0” silk suture^5^ was placed to press the graft on the root surface.

### Postoperative care

All patients assumed 2 g/day amoxicillin + clavulanic acid^6^ for 6 days; 400 mg tablets of ibuprofen^7^ was suggested to be taken only if the patient experienced significant pain.

Patients were prescribed 0.2% Chlorhexidine rinses twice daily,^8^ for 3 weeks.

Sutures were removed on the14th day. Gentle brushing with a soft toothbrush and interdental brushing were recommended starting 2 weeks after suture removal. Additionally, patients used a 1% chlorhexidine gel^8^ twice daily. Patients received weekly supragingival hygiene and motivational reinforcement for 6 weeks. Recall sessions were scheduled every 3 months during the first year and every 6 months thereafter.

### Statistical analysis

CRC was analyzed using Fisher's exact test and Bayesian logistic regression [18]. RC% and all clinical parameters were modeled using linear regression. VAS outcomes, DH test results, analgesic intake, RES and PRES scores were analyzed using cumulative logistic models, DWB and OTS using Logistic Regression. CWE was modeled using Cox's Proportional Hazard model. Tree models were used for model selection. The statistical analysis was performed using R software [19].

## Results

### Study population

All 40 patients (28 women; 38.2 ± 0.6 years old, range 21–63) completed the 12-month follow-up. The study included twenty-five incisors, eleven canines and four premolars (upper and lower), with no significant differences in tooth distribution between the groups (SM 3). No patient reported complications. FMPS and FMBS were maintained at < 15% throughout the study, with no differences between the groups.

### Main outcome, clinical treatment outcomes

Nineteen patients in the FTPGT group achieved CRC compared to 12 in the CAF + SCTG group, with a 0.35% CRC difference and OR = 11.92, *p* < 0.0196 (Fisher's test) or *p* = 0.026 (Bayesian Logistic Regression).

On average, the FTPGT treatment obtained a greater RC% by 12.71 points ± 3.82, *p* < 0.002, according to a design-based inference (ANOVA). It showed less GR (−0.7 mm ± 0.19, *p* < 0.001), a greater gain in CAL (0.65 mm ± 0.20, *p* < 0.002), GT (0.99 ± 0.27, *p* < 0.001) and KTW (2.95 ± 0.5, *p* < 0.001), while PD was similar (0.054 mm ± 0.15, *p* = 0.74).

Three cases having RC% > 100 appeared as outliers. Sensitivity analysis excluding these cases confirmed the results (SM 5–8).

**Fig. 3:**
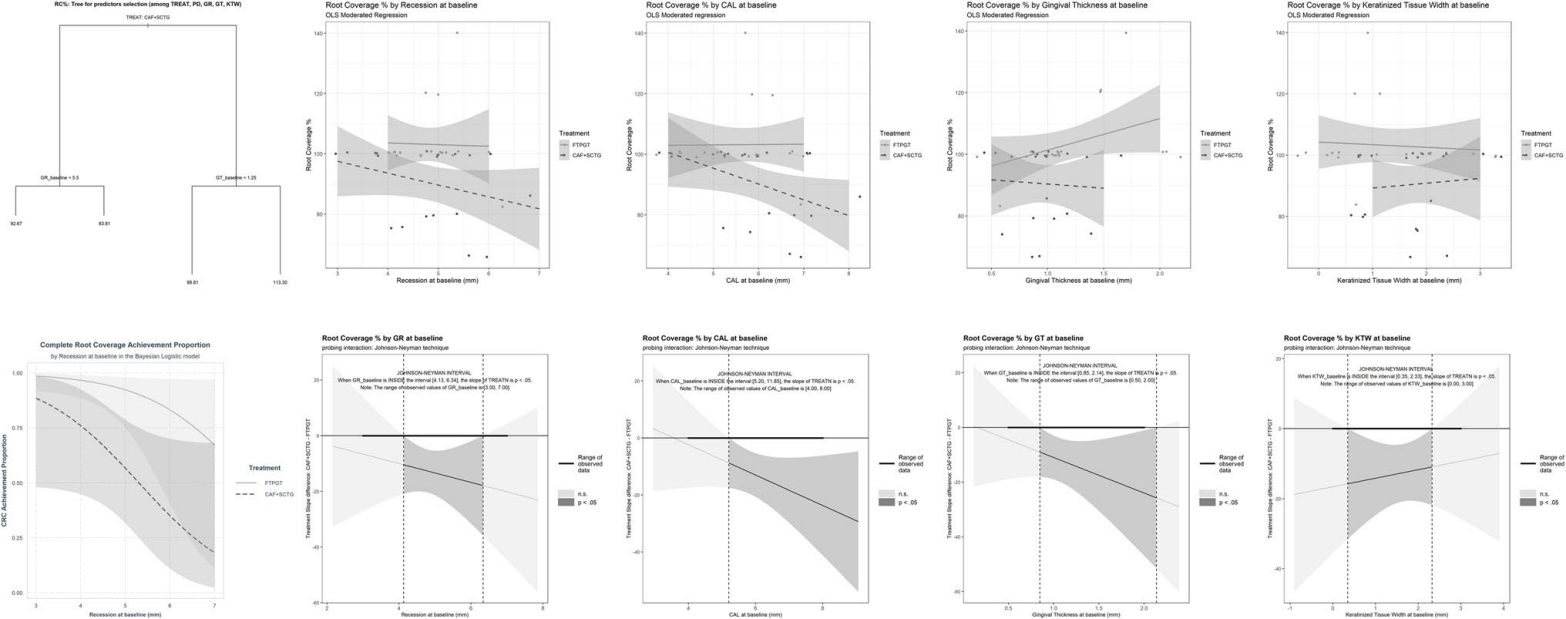
First column: Tree model for predictors selection (from TREAT, PD, GR, GT, KTW) of RC%; Bayesian Logistic Regression of the resulting model (CRC ~ GR at baseline + TREAT) on the binary CRC outcome. Column 2–5: ordinary least squares moderated regression model on the RC% for each one of the 4 parameters– i.e. 2^nd^ column: RC% ~ GR_baseline * TREAT with its Johnson-Neyman significance region (4.13 to 6.34)

Recalling that the sample-size had not been computed for this outcome (let alone for an interaction term, probably requiring four times that for main effects [20]), all the parameters at T0 were tested as covariates resulting inconclusive, although appeared to appreciably improve predictions. Their impact as moderators is shown in Fig. 3 columns 2–5 and SM 6 and reported and discussed in SM 10–11.

In SM 4, at the bottom, and SM 9 we provided a sense of the impact of the full palatal thickness gathered for the graft (non-measured just after its drawing) by analyzing RC% by GT change, hypothizing its variance represents a composite of the variance of the graft thickness and that of the postoperative shrinkage measured at 1 year follow-up.

### Patient-reported outcomes

**Fig. 4:**
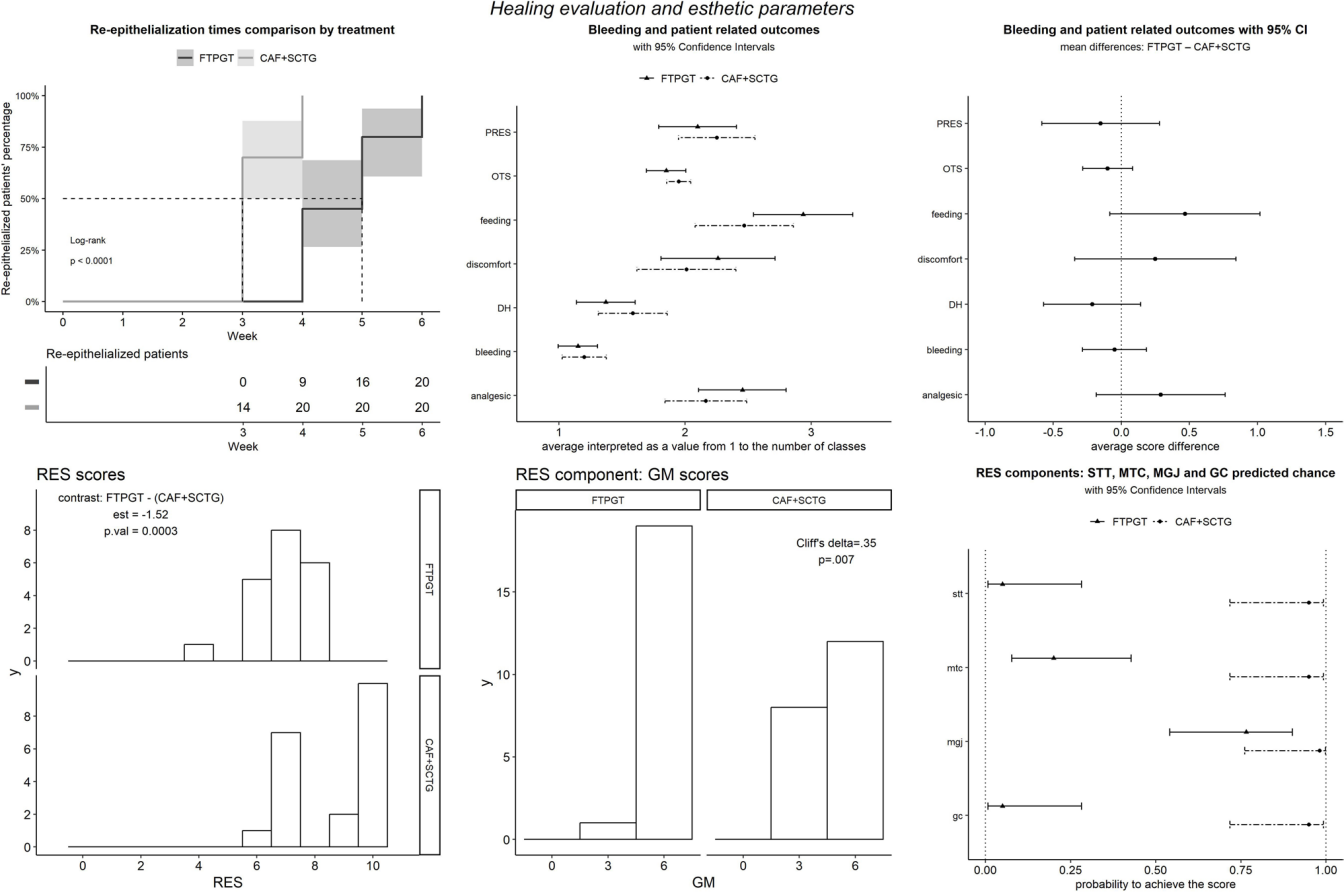
From top-left to bottom-right: re-epithelialization time comparison, healing parameters means and 95% CI first and means and 95% CI of the treatment differences, RES scores histograms, GM scores histograms and probability of success in getting the partial esthetic scores. NB: the"averages"depicted in the graphs relative to ordinal models are not arithmetic averages of the ordinal values but are obtained by means of the"mean.class"mode of the emmeans R package, which is the average of the probability distributions as probabilities of the integers. Please, see: https://cran.r-project.org/web/packages/emmeans/vignettes/sophisticated.html#ordinal

Most of the differences between FTPGT and CAF + SCTG groups were smaller than half a point (Fig. 4): analgesic tablets consumption = 0.29 ± 0.24, *p* = 0.24 (observed median difference = 0.5 tablets), D = 0.25 ± 0.3, *p* = 0.41, CFH = 0.47 ± 0.28, *p* = 0.11, PRES = 0.152 ± 0.22, *p* = 0.49 and DH reduction = 0.306 ± 0.159, *p* = 0.055. The OTS difference scored −1.2 ± 1.2, OR = 0.3 (0.03 to 3.15), *p* = 0.42.

### Esthetic outcomes

Regarding RES scores, the CAF + SCTG treatment outperformed the FTPGT by 1.52 ± 0.421, *p* < 0.0003. Among the 5 variables that make up RES, GM showed better results in the FTPGT group (Cliff's delta = 0.35, *p* = 0.007), while MGJ was inconclusive (Fig. 4).

### Palatal wound healing outcomes

FTPGT was significantly slower to heal, HR = 0.13 ± 0.06, *p* < 0.0001: at the fourth week 9 FTPGT patients had healed compared to 20 in the CAF + SCTG group (Fig. 4). All patients recovered by the sixth week. Postoperative bleeding episodes were comparable, OR = 1.4 (0.20 to 11.13), *p* = 0.68. Sensitivity analysis available in SM 12 (Table 1).

**Table 1:** Clinical parameters observed mean ± SD in millimetres (*n* = 20 in each group)

Parameter	Treatment	Baseline mean ± SD (95% CI)	12 months mean ± SD (95% CI)	Baseline-12 months mean (95% CI)	Within- group differences
PD	FTPGT	1 ± 0.56 (0.74 to 1.26)	1.25 ± 0.55 (0.99 to 1.51)	0.25 ± 0.79 (−0.12 to 0.62)	NS
	CAF + SCTG	1.15 ± 0.37 (0.98 to 1.32)	1.25 ± 0.44 (1.04 to 1.46)	0.1 ± 0.55 (−0.16 to 0.36)	NS
	Between Groups Difference	NS	NS	NS	
CAL	FTPGT	5.7 ± 1.03 (5.22 to 6.18)	1.1 ± 0.55 (0.84 to 1.36)	4.6 ± 1.19 (4.04 to 5.16)	< 0.001
	CAF + SCTG	5.95 ± 1.1 (5.44 to 6.46)	1.75 ± 0.72 (1.41 to 2.09)	4.15 ± 0.93 (3.71 to 4.59)	< 0.001
	Between Groups Difference	NS	*p* < 0.003	NS	
GR	FTPGT	4.8 ± 0.62 (4.51 to 5.09)	−0.15 ± 0.59 (−0.42 to 0.12)	−4.95 ± 0.83 (−5.34 to −4.56)	< 0.001
	CAF + SCTG	4.8 ± 1.06 (4.31 to 5.29)	0.5 ± 0.69 (0.18 to 0.82)	−4.25 ± 0.91 (−4.68 to −3.82)	< 0.001
	Between Groups Difference	NS	*p* < 0.003	*p* < 0.015	
KTW	FTPGT	1.3 ± 1.08 (0.79 to 1.81)	6.25 ± 0.97 (5.8 to 6.7)	4.3 ± 2.18 (3.28 to 5.32)	< 0.001
	CAF + SCTG	1.75 ± 0.64 (1.45 to 2.05)	3.1 ± 0.79 (2.73 to 3.47)	1.35 ± 0.49 (1.12 to 1.58)	< 0.001
	Between Groups Difference	NS	*p* < 0.001	*p* < 0.001	
GT	FTPGT	1.18 ± 0.44 (0.97 to 1.38)	2.95 ± 1.3 (2.34 to 3.56)	1.77 ± 1.12 (1.25 to 2.3)	< 0.001
	CAF + SCTG	0.98 ± 0.3 (0.83 to 1.12)	1.76 ± 0.37 (1.59 to 1.93)	0.78 ± 0.45 (0.57 to 1)	< 0.001
	Between Groups Difference	NS	*p* < 0.001	*p* < 0.02	
Recessions obtaining Complete Root Coverage	FTPGT		19 (95%)		
	CAF + SCTG		12 (60%) *p* < 0.004		
Root Coverage %	FTPGT		103.17 ± 11.42 (97.82 to 108.51)		
	CAF + SCTG		90.45 ± 12.68 (84.52 to 96.38)		
	Between Groups Difference		*p* < 0.002		
RES score	FTPGT		7 (6 to 8)		
	CAF + SCTG		9.50 (7 to 10)		
	Between Groups Difference		*p* = 0.0003		
		Healing week			
		third	fourth	fifth	sixth
Re-epithelialized patients per week	FTPGT	-	9	16	20
	CAF + SCTG	14	20	20	20
Bleeding	FTPGT		3 (15%)		
	CAF + SCTG		4 (20%)		
	Between Groups Difference		NS		
Pain (VAS)	FTPGT		3 (2 to 4)		
	CAF + SCTG		3 (2 to 3.25)		
	Between Groups Difference		NS		
Analgesic tablet consumption	FTPGT		3.5 (3 to 4)		
	CAF + SCTG		3 (3 to 4)		
	Between Groups Difference		NS		
Feeding Habit Changes (VAS)	FTPGT		4 (3 to 4.25)		
	CAF + SCTG		3.5 (3 to 4)		
	Between Groups Difference		NS		
Dentine Hypersensitivity	FTPGT	2 (2 to 3)	0 (0 to 1)		
	CAF + SCTG	2 (2 to 3)	0.5 (0 to 1)		
	Between Groups Difference	NS	NS		
Patient Related Esthetic Score	FTPGT		9 (9 to 10)		
	CAF + SCTG		9 (9 to 10)		
	Between Groups Difference		NS		
Overall Treatment Satisfaction	FTPGT		17 (85%)		
	CAF + SCTG		19 (95%)		
	Between Groups Difference		NS		

NB. Continuous variables are reported as specified in the header (mean ± SD) (95% CI), binary as success frequency (%) while ordinal as median (1st to 3rd quartile). The Complete Root Coverage proportions at follow-up were analysed by means of Fisher's exact test. All the other tests of significance, inappropriate since referring to the populations whilst here we are interested in the samples, are included because they are customary

## Discussion

### Principal findings

Both CAF + SCTG and FTPGT treatments significantly improved clinical conditions; however, FTPGT achieved a significantly greater CRC (Table 2, Fig. 3). The primary factor responsible for this result may be the greater thickness of the graft, which, in its central part, equals the entire thickness of the soft palatal tissues. The Literature on different surgical techniques reports that the thicker the tissue placed on the exposed root, the more likely it is to obtain CRC [21–23]. This may be due to the greater chance of survival for thick tissues placed over an avascular surface, such as the exposed root [24]. In fact, thick tissues have a greater abundance of vessels [22], facilitating its revascularization. Better clinical outcome may result from a very thick graft, which captures an abundant blood supply from the adjacent tissues [25]. This is further facilitated by the absence of releasing incisions at the recipient site in the FTPGT, which helps preserve the blood supply to the graft [8]. Furthermore, the free graft used in the FTPGT includes the periosteum whose inner layer is rich in stem cells with great regenerative potential, capable of producing new cementum and periodontal ligament [26]. All these observations may help explain why the FTPGT group, in some cases, achieved root coverage exceeding 100%, with the gingival margin extending well beyond the CEJ.

**Table 2:** FTPGT– CAF + SCTG differences between estimated mean changes from baseline to 12 months (*n* = 20 in each group) for the clinical parameters and between differences at follow-up for the remaining ones

Parameter	Mean difference ± SE (95% CI)	OR ± delta SE (95% CI)	Log (HR) ± SE (95% CI)	HR ± delta SE (95% CI)	*p*-value
PD (in mm)	0.054 ± 0.15 (−0.25 to 0.36)				0.74
CAL gain (in mm)	0.65 ± 0.20 (0.26 to 1.05)				< 0.002**
GR (in mm)	- 0.7 ± 0.19 (−1.09 to −0.31)				< 0.001***
KTW (in mm)	2.95 ± 0.5 (1.94 to 3.96)				< 0.001***
GT (in mm)	0.99 ± 0.27 (0.44 to 1.54)				< 0.001**
CRC Fraction Fisher's test Bayes Logistic Regression LR test	0.35 (0.065 to 0.63)	11.92 (1.32 to 588.83)			0.019* < 0.006**
RC %	12.71 ± 3.82 (4.99 to 20.44)				< 0.002**
RES score	−1.52 ± 0.421 (−2.34 to −0.7)				0.0003***
Re-epithelialization time (in weeks)	1.45 (1.059 to 1.841)		−2.05 ± 0.43 (−2.89 to −1.2)	0.13 ± 0.06 (0.06 to 0.30)	< 0.0001*** < 0.0001***
Bleeding	−0.05 ± 0.12 (−0.285 to 0.185)	1.40 (0.20 to 11.13)			0.68
Discomfort (VAS)	0.25 ± 0.30 (−0.34 to 0.84)				0.41
Analgesic tablets consumption	0.29 ± 0.24 (−0.18 to 0.76)				0.24
Feeding Habit Changes (VAS)	0.47 ± 0.28 (−0.08 to 1.02)				0.11
Dentine Hypersensitivity	0.306 ± 0.159 (−0.007 to 0.62)				0.055
Patient related esthetic score	−0.152 ± 0.22 (−0.58 to 0.28)				0.49
Overall treatment satisfaction	−1.20 ± 1.20 (−3.77 to 1.35)	0.3 ± 0.36 (0.03 to 3.15)			0.42

NB. Bleeding and OTS were analysed by means of Logistic Regression. The outcomes in VA-Scale along with Analgesic usage, Schiff test, RES scores and Patient Related Esthetic scores were analysed by means of cumulative logistic models and reported in a latent-variable scale (R package"emmeans"with mode = mean.class), while Re-epithelialization time using Cox's proportional hazard model. All remaining parameters were analysed by means of linear models. The RC% model is interactive. The probing of the interaction with the J-N significance region is shown in the bottom-right plot of Fig. 3

### Agreements and disagreements with previous findings

This is the first study comparing the FTPGT with other surgical techniques, making direct comparison with previous studies impossible. However, in a previous case series report, Paolantonio et al. observed CRC in 14 of the 15 treated teeth (93,3% CRC); in this study, the FTPGT group achieved a very similar result (95% CRC) [8]. Although our active comparator, CAF + SCTG, is recently reiterated as the gold standard for covering exposed roots [9]. It is interesting to note that the performance of the CAF + SCTG group in this study is very similar to that reported in various meta-analyses [6, 9]. A recent meta-analysis by Chambrone et al., showed an average CRC, 0.72% (0.57 to 0.83) for the CAF + SCTG technique, slightly higher than ours (SM 13) [9]. In evaluating CRC, we used the definition criterion by Pini Prato et al. [7], which is based on histological considerations and is more restrictive than the one used in many studies, whereby a CRC is achieved when the gingival margin is at the level of the CEJ.

### Secondary outcomes

The superior results obtained by the FTPGT group in improving CAL, GT and RC% can be explained by the same biological reasons that justify a better CRC. The great thickness of the graft in FTPGT also explains the follow-up result of a greater GT compared to the CAF + SCTG group.

The greater increase in KTW in the FTPGT group is explained by the fact that the graft includes a quantity of keratinized epithelium equal to the depth of GR + 1 mm while the keratinization of the flap covering the SCTG is only partially influenced by the underlying transplant [27–29].

Patient reported outcomes did not differ between the two groups.

It is surprising that FTPGT, requiring a more invasive procedure for graft harvesting, doesn’t cause greater discomfort, changes in chewing habits, or the need for more painkillers. Zucchelli et al. hypothesized that postoperative discomfort may depend on the thickness of the residual soft tissue on the palatal bone [30]. In the case of FTPGT no connective tissue remains to cover the bone, and the periosteum is also removed. It could be speculated that the post-operative discomfort has been limited by the complete removal of the nociceptive nerve terminations that don’t remain exposed but are taken away together with the graft, post-operative pain deriving only from the section of the small nerve fibres in the corium. Furthermore, the lack of greater morbidity in the FTPGT group may be due to the protection of the wounded area by a haemostatic collagen sheet, which could have minimized post-operative pain.

PRES didn’t differ between the two groups; patient satisfaction is not always dependent on CRC, which was better in the FTPGT group, or the other clinical parameters included in the RES, with higher scores in the CAF + SCTG group, critically evaluated by a Professional [31].

Therefore, the similar OTS score detected in the 2 groups can be explained by the concomitant similarity of morbidity parameters and PRES values.

The similar DH scores improvement follows the good RC% obtained in both groups; probably, the extent of difference in CRC doesn’t influence this patient related outcome.

The esthetic outcome is of paramount importance in periodontal plastic surgery. Although CRC has a significant impact on the RES index, the CAF + SCTG group performed better overall than the FTPGT group, despite the latter achieving higher CRC. This is due to the less favorable results obtained by FTPGT in other components of the RES, such as marginal contour, gingival color, and scar or keloid appearance. The greater thickness of the graft, which allows FTPGT to excel in CRC, also limits its esthetic evaluation. In fact, the large dimensions of the graft do not favor esthetics, and thin or even absent grafts associated with CAF produce better esthetic results [32, 33]

As expected, the greater volume of tissue removed from the palate explains the longer healing time necessary for the FTPGT group (Tables 1, 2) [8]. However, this was not associated with more frequent bleeding episodes or other complications compared to the CAF + SCTG group.

### Clinical implications

Compared to the"gold standard"technique for covering exposed roots, the FTPGT has shown a higher probability of achieving CRC and producing a greater increase in KTW. Therefore, FTPGT may be suggested for the treatment of deep single GRs associated with a lack of keratinized tissue such as Miller's second class. However, FTPGT produces less esthetic performance with worse blending in color, regularity of the gingival profile, and potential for scarring, making it less suitable for areas of esthetic relevance.

Miller's second class GRs in the lower arch should be given as a clear example of a site where the technique would be applicable.

Furthermore, although there are no clinical consequences, the healing time of the donor site is significantly longer for FTPGT.

### Limitations of the study

Firstly, the mean GT_T0_ value is in both groups higher than the value (0.8 mm) under which a graft is suggested [34]. Whether these results will be confirmed in the presence of a thin gingival biotype remains to be evaluated.

Furthermore, this study did not evaluate the surgical chair time, and it should be considered that the graft harvesting procedure in FTPGT is more time-consuming.

In this study, GT was measured using a simple invasive clinical method. Recent studies have quantified GT with more advanced technological methods, such as non-invasive radiographic or ultrasonic techniques [35, 36]. However, the method we used cannot be considered obsolete, as it has been demonstrated that digital measurement of GT is comparable to direct clinical assessments performed with transgingival horizontal probing using an endodontic instrument [35, 37, 38], while some authors consider direct transgingival measurement as the most accurate method [39].

Furthermore, the effect modification exerted by tooth position (upper or lower arch; tooth type) was not assessed in this study because we calculated a sample size sufficient to include only one predictor (treatment). Indeed, the stratification needed to evaluate effect modification by arch and position requires substantially larger samples to avoid sparse data, so we postponed the assessment of effect modification by tooth position to a future study with a substantially larger sample.

Finally, these treatments break the examiner’s blind.

## Conclusions

Within the limitation of our study, we conclude that

- FTPGT is more effective than CAF + SCTG in achieving CRC;
- This is particular true for deep single recessions;
- FTPGT increases GT and KTW more than CAF + SCTG;
- CAF + SCTG is associated with better esthetic results;
- Complete healing of the graft donor site takes longer with FTPGT
- Patient-related outcomes don’t significantly differ between the two techniques.
- These results need to be confirmed in cases with very thin (< 0.8 mm) GT at baseline.

## Supplementary Information

Below is the link to the electronic supplementary material.

Supplementary file1 (DOCX 15927 KB)

## Data Availability

The research data supporting the results of this manuscript are included within the manuscript itself.

## Footnotes

1 Hu-Friedy, Chicago, IL.

2 Komet Italia S.r.l., Milano, Italy.

3 Fine Science Tools Inc., Fosters City, CA, USA.

4 Ethicon, Johnson & Johnson, Pomezia, Italy.

5 Ethicon, Johnson & Johnson, Pomezia, Italy.

6 Augmentin, SmithKline Beecham, Milan, Italy.

7 Nurofen Express 400 mg, Reckitt Benckiser Group, Slough, Berkshire, UK.

8 Curasept S.P.A., Saronno, Italy.
