# Supplementary material for: Clinical comparison of two surgical techniques in obtaining complete root coverage of single RT1 gingival recessions
Source: Clin Oral Investig. 2025 Sep 10;29(10):444. doi: 10.1007/s00784-025-06491-2 (PMC12423173; doi:10.1007/s00784-025-06491-2)
Supplement: Supplementary file 1 — Supplementary file1 (DOCX 15927 KB) [file 784_2025_6491_MOESM1_ESM.docx]

Supplementary material

Supplementary Material 1


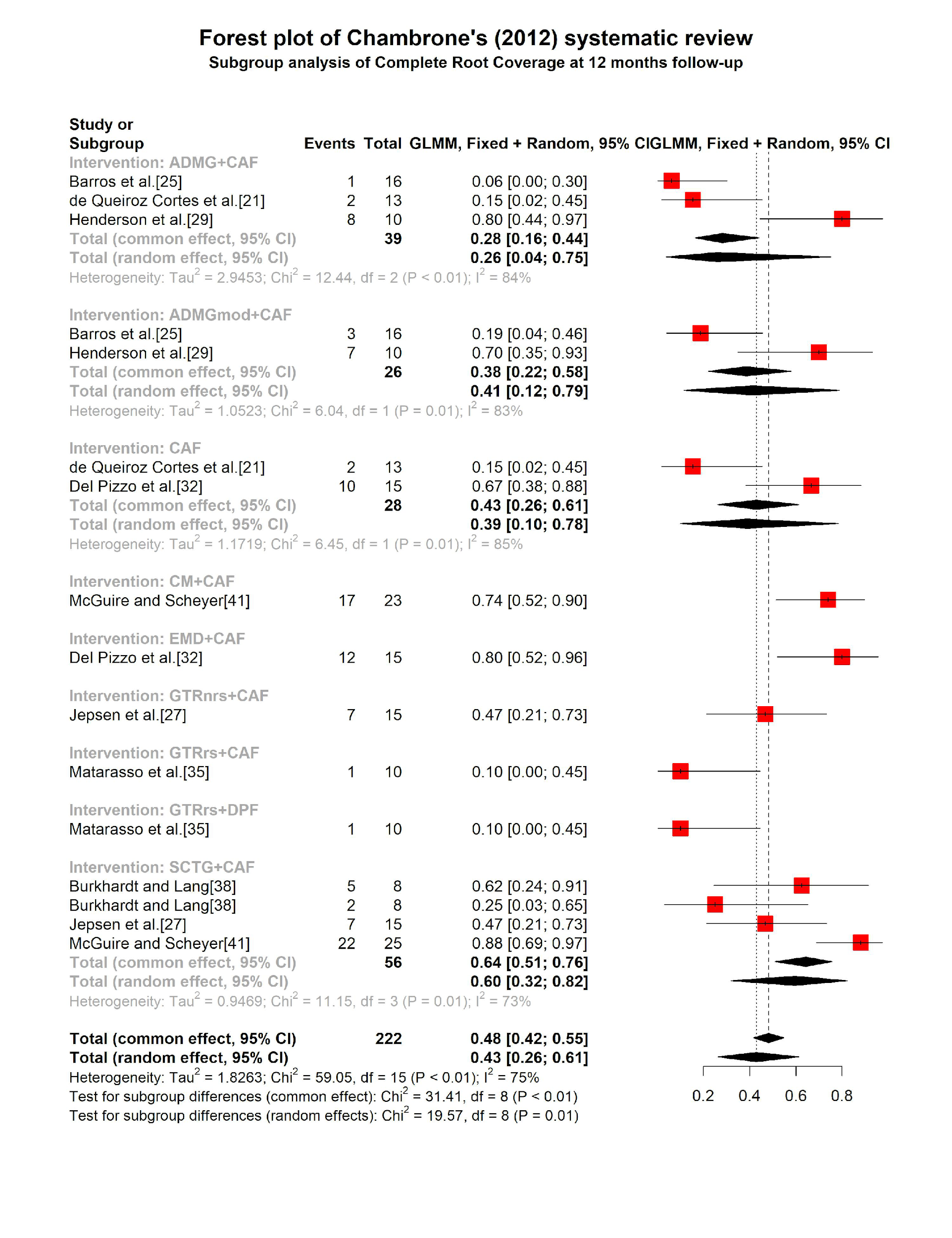


Meta-analysis of Chambrone's (2012) systematic review studies on CAF+SCTG reporting CRC at a 12 months follow-up, with subgroup analysis.

Supplementary Material 2

***Sample size calculation***

According to Agresti 1996 *An Introduction to Categorical Data Analysis*, p.161:

n1 = n2 = (zα/2 + zβ)2 [π1(1 − π1) + π2(1 − π2)] / (π1 − π2)2

(qnorm(.025)+qnorm(.2))^2 * (.95*(1-.95) + .6*(1-.6)) / (.95-.6)^2

[1] 18.42084

Supplementary Material 3

**Demographic parameters at baseline.**

| **Parameter** | **Treatment** | **Baseline mean ± SD**  **(95% CI)** |
| --- | --- | --- |
| **Age** | FTPGT | 43.95 ± 13.08  (37.83 to 50.07) |
|  | CAF+SCTG | 42 ± 15.13  (34.92 to 49.08) |
|  | Betweeen Groups Difference | NS |
| **Gender (Female)** | FTPGT | 13 (65%) |
|  | CAF+SCTG | 14 (70%) |
|  | Betweeen Groups Difference | NS |
| **Tooth type** | FTPGT | IC:1(5%), SC:6(30%), II:5 (25%), SI:4(20%), IP:2(10%), SP:2(10%) |
|  | CAF+SCTG | IC:2(10%), SC:4(20%), II:7 (35%), SI:4(20%), IP:1(5%), SP:2(10%) |
|  | Betweeen Groups Difference | NS |
| **FMPS** | FTPGT | 11.14 ± 2.31  (10.06 to 12.22) |
|  | CAF+SCTG | 10.85 ± 1.97  (9.93 to 11.77) |
|  | Betweeen Groups Difference | NS |
| **FMBS** | FTPGT | 8.61 ± 2.68  (7.36 to 9.87) |
|  | CAF+SCTG | 8.87 ± 2.4  (7.74 to 9.99) |
|  | Betweeen Groups Difference | NS |

Abbreviations: SD, standard deviation; CI, confidence interval; NS, non-significant; FTPGT, full-thickness palatal graft technique; CAF, coronally advanced flap; SCTG, sub-epithelial connective tissue graft; IC, inferior canine; SC, superior canine; II, inferior incisor; SI, superior incisor; IP, inferior premolar; SP, superior premolar; FMPS, full-mouth plaque score; FMBS, full-mouth bleeding score.

Supplementary Material 4

**Exploration of the role of the possible outliers**

A visual sensitivity analysis comparing the treatments is shown below. The slope for the FTPGT group is calculated using the full sample and various reduced samples. These reduced samples exclude, either partially or completely, the three cases with RC% > 100%, the case not achieving CRC, or the three cases with the largest GT at baseline (2 mm).


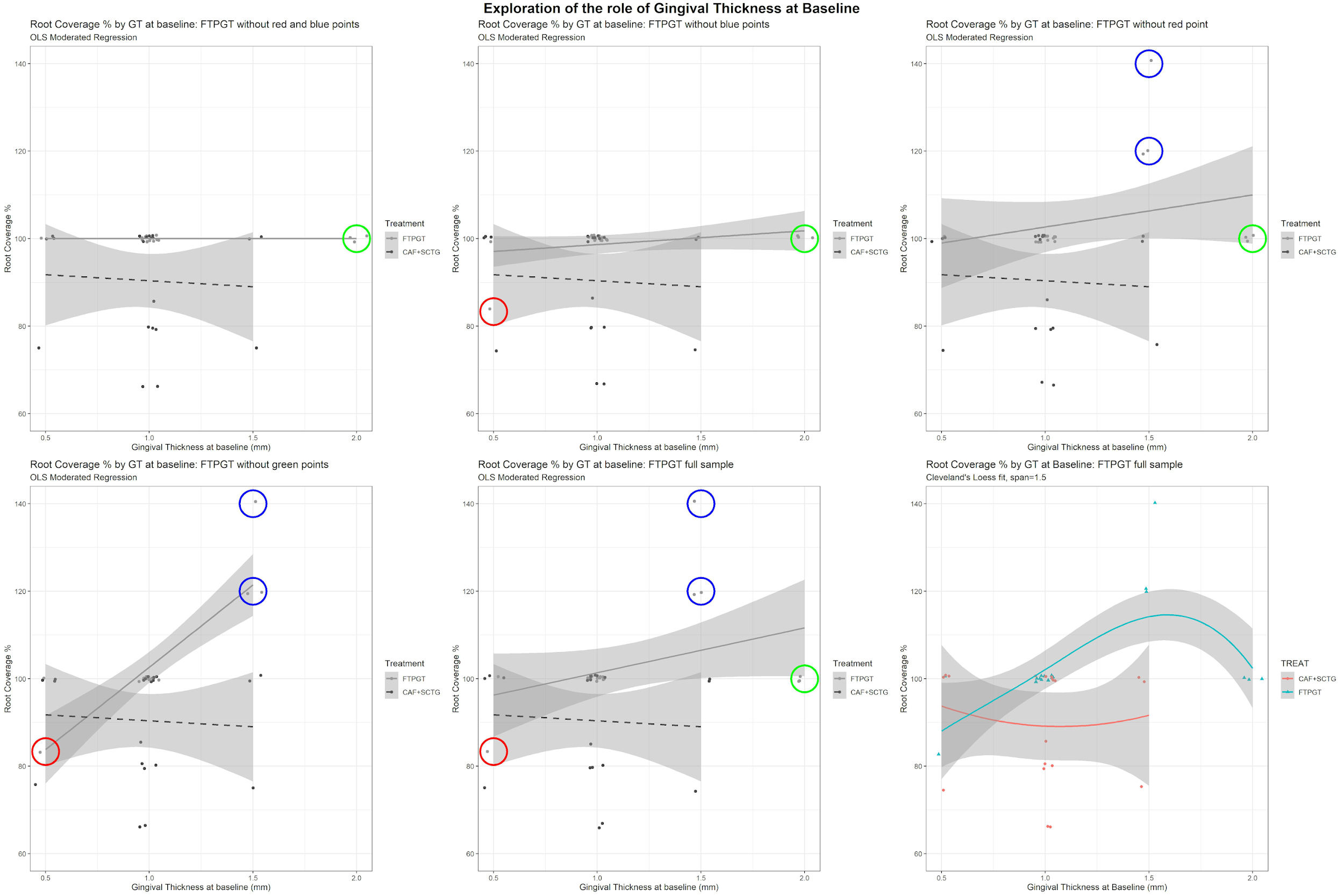


First, the rough flatness of CAF+SCTG is determined by the 8 non-CRC cases having a symmetrical GT_T0_ distribution around the mean (six of them are on the 1 mm median, while the remaining two are half a millimetre on opposite sides).

Conversely, the slope of FTPGT would be exactly zero if one case with RC% < 100 (83%) and three cases with RC% > 100% (two cases at 120% and one case at 140%) were excluded, while retaining the three cases with RC% = 100% and GT at baseline of 2 mm (top-left, green circle). Re-adding only the case with RC% < 100% (red circle), the slope is minimal (top-centre). It becomes steeper by including only the three cases with RC% > 100% (blue circles, top-right). Including all four cases with RC% ≠ 100% while excluding the three cases with 2 mm GT at baseline results in a maximal slope (bottom-left), while the complete sample returns to a similar situation as before (bottom-centre). These observation allow us to appreciate the extent the final result depends on the leverage exerted by 1 non-CRC case (83.3%) at GT_T0_= 0.5 and the three y-cases > 100% at GT_T0_ = 1.5, which is somehow mitigated by the three cases with maximal GT_T0_ (2 mm). The last plot (bottom-right) shows what the regression line slope would be if the linearity constraint was removed (left-side slope almost as steep as bottom-left plot).

The next figure shows the influence of the parameters at baseline CAL, GT and KTW on the RC% according to Cleveland's Loess estimator. The first row represents a full-sample, and the second row shows the results after removing the three potential outliers with RC% > 100% from the FTPGT group. The last row displays the linear interactive models for the purged samples.

The hump in the FTPGT treatment estimates disappears once the possible outliers are removed. The curves appear almost flat (second row) and closely approximated by the straight lines of the linear models (third row).

The fact that the ANOVA model assumptions result acceptable

> COP.ano<-lm(PERC_COP~TREAT); summary(gvlma(COP.ano))

Call:

lm(formula = PERC_COP ~ TREAT)

Residuals:

Min 1Q Median 3Q Max

-23.792 -3.167 -3.167 9.549 36.834

Coefficients:

Estimate Std. Error t value Pr(>|t|)

(Intercept) 90.452 2.698 33.528 < 0.0000000000000002 ***

TREATFull Thickness 12.715 3.815 3.333 0.00193 **

---

Signif. codes: 0 ‘***’ 0.001 ‘**’ 0.01 ‘*’ 0.05 ‘.’ 0.1 ‘ ’ 1

Residual standard error: 12.06 on 38 degrees of freedom

Multiple R-squared: 0.2262, Adjusted R-squared: 0.2058

F-statistic: 11.11 on 1 and 38 DF, p-value: 0.001926

ASSESSMENT OF THE LINEAR MODEL ASSUMPTIONS

USING THE GLOBAL TEST ON 4 DEGREES-OF-FREEDOM:

Level of Significance = 0.05

Call:

gvlma(x = COP.ano)

Value p-value Decision

Global Stat 2.585473176814046870 0.6294 Assumptions acceptable.

Skewness 0.892811447472015463 0.3447 Assumptions acceptable.

Kurtosis 1.619611210119610512 0.2031 Assumptions acceptable.

Link Function 0.000000000000004836 1.0000 Assumptions acceptable.

Heteroscedasticity 0.073050519222416024 0.7869 Assumptions acceptable.

might depend on insufficient power. The functional form of the purged sample appears more plausible and generalizable.


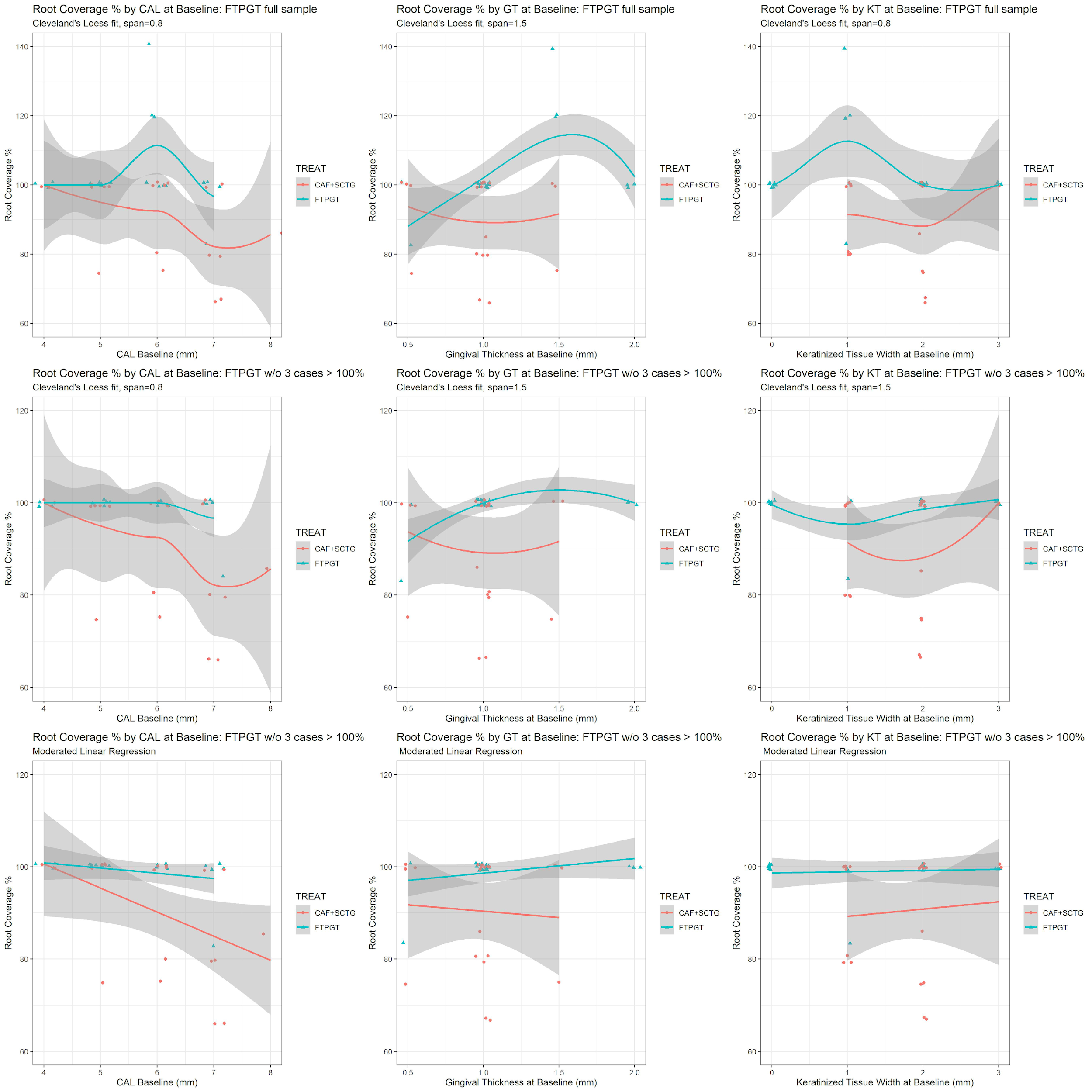
The question arises whether the outliers are due to recording errors or if they are genuine outlying observations. The first point to consider is that they are not impossible outcomes.

Their treatment in the analysis should probably differ based on whether they are due to gross errors or to real but outlying observations. In the first case they should be excluded from the analysis. In the second case, since they at least achieved CRC, an analysis that winsorizes them to 100 might be more realistic to prevent Type II errors. Both analyses are available in SM 5, 7 and 8 (of which the SM 6 is the full sample reference): the results are not substantively different.

Moreover, a visual analysis of the model **RC% = GT_T0_ + Treatment + GT_T0_ :Treatment** with Cleveland's loess smoother performed after excluding the three outliers greater than 100% RC was conducted. This explains why, in the tree model, GT_T0_ is never a useful predictor for the CAF+SCTG treatment while for FTPGT it is so only if the outliers are included.

~~
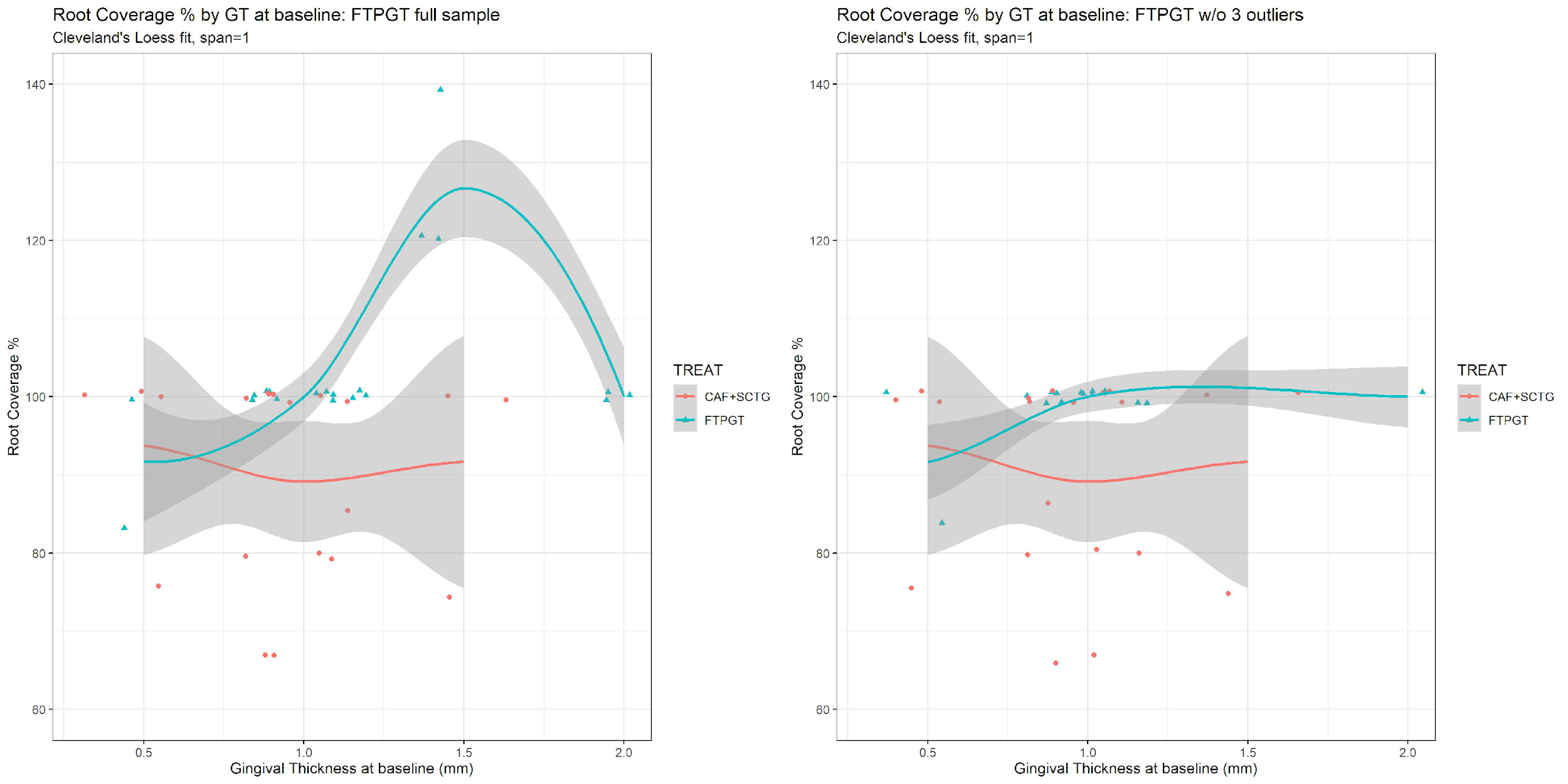
~~

It is worth noting that the 3 outliers had a quite thick gingiva at baseline (1.5 mm) although not the thickest: three different cases had a larger thickness (2 mm) but did not obtain the largest thickness at follow-up (respectively 4, 3.5, and 2 mm).

which(GT_baseline>1.5)

[1] 5 11 18

TK_POST[c(5,11,18)] **# GT at follow-up in the 3 cases having GT at baseline > 1.5 mm**

[1] 4.0 3.5 2.0

which(GT_baseline==1.5) **#** **all the cases having GT at baseline = 1.5 mm**

[1] 1 8 15 30 31 33

**# GT at follow-up in all the cases having GT at baseline = 1.5 mm**

TK_POST[which(GT_baseline==1.5)]

[1] 5.5 5.5 5.5 2.5 1.5 1.5


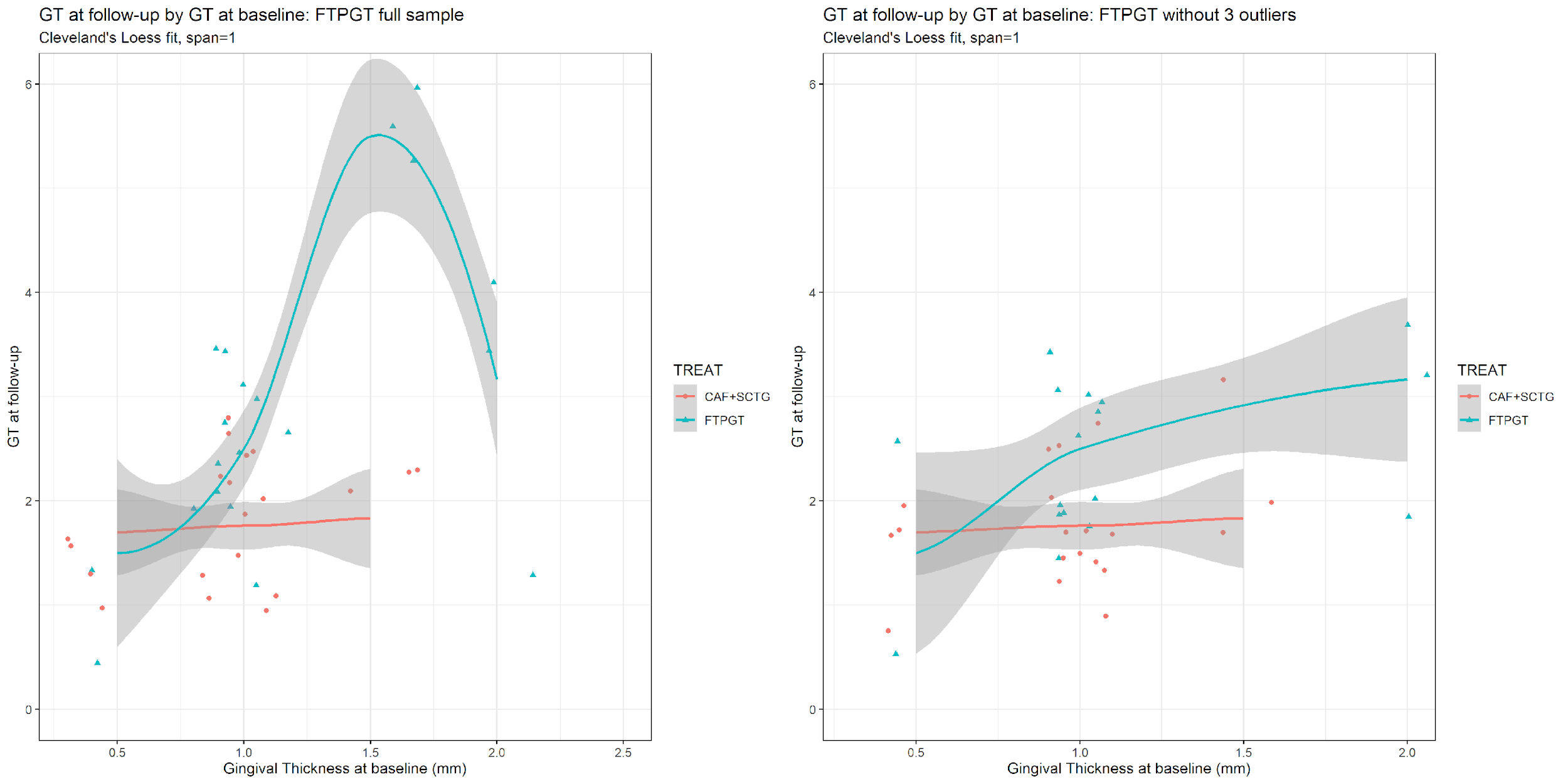


This observation led us to test the variance of the thickness variation between baseline and 1 year follow-up, which is a composite of the variance of the graft thickness added to that of the postoperative shrinkage measured at 1 year follow-up. As expected, FTPGT has 6 times the variance of CAF+SCTG.

> tapply(TK_CH, TREAT2, var)

FTPGT CAF+SCTG

1.2493421 0.2066053

> var.test(TK_CH~ TREAT2)

F test to compare two variances

data: TK_CH by TREAT2

F = 6.047, num df = 19, denom df = 19, p-value = 0.0002606

alternative hypothesis: true ratio of variances is not equal to 1

95 percent confidence interval:

2.393476 15.277450

sample estimates:

ratio of variances

6.047

**# Purged sample**

> with(FullT[-c(5,11,18),], tapply(TK_CH, TREAT2, var))

FTPGT CAF+SCTG

1.2665441 0.2066053

> with(FullT[-c(5,11,18),], var.test(TK_CH~ TREAT2))

F test to compare two variances

data: TK_CH by TREAT2

F = 6.1303, num df = 16, denom df = 19, p-value = 0.000312

alternative hypothesis: true ratio of variances is not equal to 1

95 percent confidence interval:

2.366299 16.539619

sample estimates:

ratio of variances

6.130261

A more extreme variances ratio is shown by the KTW parameter.

> tapply(KT_CH, TREAT2, var)

FTPGT CAF+SCTG

4.7473684 0.2394737

> var.test(KT_CH~ TREAT2)

F test to compare two variances

data: KT_CH by TREAT2

F = 19.824, num df = 19, denom df = 19, p-value = 0.00000001933

alternative hypothesis: true ratio of variances is not equal to 1

95 percent confidence interval:

7.84665 50.08481

sample estimates:

ratio of variances

19.82418

**# Purged sample**

> with(FullT[-c(5,11,18),], tapply(KT_CH, TREAT2, var))

FTPGT CAF+SCTG

5.2426471 0.2394737

> with(FullT[-c(5,11,18),], var.test(KT_CH~ TREAT2))

F test to compare two variances

data: KT_CH by TREAT2

F = 21.892, num df = 16, denom df = 19, p-value = 0.00000001232

alternative hypothesis: true ratio of variances is not equal to 1

95 percent confidence interval:

8.450521 59.066248

sample estimates:

ratio of variances

21.89237

Supplementary material 5

**SENSITIVITY ANALYSIS excluding the three outlying cases showing RC%>100**

We checked the robustness of the result (the impact of removing the possible outliers) by means of a sensitivity analysis, on the main outcome and on the RC% outcome (avoiding the normality assumption - using Cliff's test and permutation test).

**# Individuating cases with RC% > 100**

which(PERC_COP>100)

[1] 1 8 15

**# SENSITIVITY ANALYSIS: MAIN OUTCOME, CRC**

with(FullT[-c(1,8,15),], chisq.test(ftable(TREAT,COP_SN), correct=FALSE)) # COP_SN stands for CRC Yes/No

Pearson's Chi-squared test

data: ftable(TREAT, COP_SN)

X-squared = 5.8108, df = 1, p-value = 0.01593

Messaggio di avvertimento:

In chisq.test(ftable(TREAT, COP_SN), correct = FALSE) :

L'approssimazione al Chi-quadrato potrebbe essere inesatta

with(FullT[-c(1,8,15),], chisq.test(ftable(TREAT,COP_SN), simulate.p.value=TRUE))

Pearson's Chi-squared test with simulated p-value (based on 2000 replicates)

data: ftable(TREAT, COP_SN)

X-squared = 5.8108, df = NA, p-value = 0.02049

with(FullT[-c(1,8,15),], fisher.test(ftable(COP_SN,TREAT)))

Fisher's Exact Test for Count Data

data: ftable(COP_SN, TREAT)

p-value = 0.02267

alternative hypothesis: true odds ratio is not equal to 1

95 percent confidence interval:

1.09978 500.72105

sample estimates:

odds ratio

10.06271

**# SENSITIVITY ANALYSIS FOR COCHRAN-MANTEL-HAENSZEL TEST**

tabella3<-with(FullT[-c(1,8,15),], table(COP_SN,TREAT,CAL));tabella3

, , CAL = 4

TREAT

COP_SN CAF+SCTG Full Thickness

NO 0 0

SI 2 3

, , CAL = 5

TREAT

COP_SN CAF+SCTG Full Thickness

NO 1 0

SI 4 5

, , CAL = 6

TREAT

COP_SN CAF+SCTG Full Thickness

NO 2 0

SI 4 4

, , CAL = 7

TREAT

COP_SN CAF+SCTG Full Thickness

NO 5 1

SI 2 4

> mantelhaen.test(tabella3, correct=FALSE)

Mantel-Haenszel chi-squared test without continuity correction

data: tabella3

Mantel-Haenszel X-squared = 5.3256, df = 1, p-value = 0.02101

alternative hypothesis: true common odds ratio is not equal to 1

95 percent confidence interval:

1.161884 272.694936

sample estimates:

common odds ratio

17.8

> DescTools::WoolfTest(tabella3)

Woolf Test on Homogeneity of Odds Ratios (no 3-Way assoc.)

data: tabella3

X-squared = 0.40803, df = 3, p-value = 0.9386

**# Sensitivity Analysis for the GR stratifying variable model**

tabella5<-with(FullT[-c(1,8,15),], table(COP_SN,TREAT,REC));tabella5

, , REC = 3

TREAT

COP_SN CAF+SCTG FTPGT

NO 0 0

SI 2 0

, , REC = 4

TREAT

COP_SN CAF+SCTG FTPGT

NO 2 0

SI 4 6

, , REC = 5

TREAT

COP_SN CAF+SCTG FTPGT

NO 3 0

SI 4 9

, , REC = 6

TREAT

COP_SN CAF+SCTG FTPGT

NO 3 1

SI 2 1

mantelhaen.test(tabella5, correct=F)

Mantel-Haenszel chi-squared test without continuity correction

data: tabella5

Mantel-Haenszel X-squared = 5.3315, df = 1, p-value = 0.02094

alternative hypothesis: true common odds ratio is not equal to 1

95 percent confidence interval:

1.004073 118.463763

sample estimates:

common odds ratio

10.90625

mantelhaen.test(tabella5, exact=T)

Exact conditional test of independence in 2 x 2 x k tables

data: tabella5

S = 8, p-value = 0.03571

alternative hypothesis: true common odds ratio is not equal to 1

95 percent confidence interval:

0.9869841 463.5854134

sample estimates:

common odds ratio

9.33329

DescTools::WoolfTest(tabella5)

Woolf Test on Homogeneity of Odds Ratios (no 3-Way assoc.)

data: tabella5

X-squared = 2.6731, df = 3, p-value = 0.4448

**# SENSITIVITY ANALYSIS FOR THE RC% outcome: design-based inference**

t.test(PERC_COP~TREAT2)

Welch Two Sample t-test

data: PERC_COP by TREAT

t = 3.3327, df = 37.592, p-value = 0.00194

alternative hypothesis: true difference in means between group Full Thickness and group CAF+SCTG is not equal to 0

95 percent confidence interval:

4.98877 20.44123

sample estimates:

mean in group Full Thickness mean in group CAF+SCTG

103.1665 90.4515

t.test(PERC_COP~TREAT, subset=-c(1,8,15))

Welch Two Sample t-test

data: PERC_COP by TREAT

t = 2.8563, df = 23.421, p-value = 0.008836

alternative hypothesis: true difference in means between group Full Thickness and group CAF+SCTG is not equal to 0

95 percent confidence interval:

2.368798 14.767025

sample estimates:

mean in group Full Thickness mean in group CAF+SCTG

99.01941 90.45150

**# ANOVA not assuming equal variances**

oneway.test(PERC_COP ~ TREAT, data=FullT[-c(1,8,15),], var.equal=FALSE)

One-way analysis of means (not assuming equal variances)

data: PERC_COP and TREAT

F = 8.1584, num df = 1.000, denom df = 23.421, p-value = 0.008836

**# Permutation test**

> coin::oneway_test(PERC_COP ~ TREAT, data=FullT, alternative="two.sided", distribution="exact")

Exact Two-Sample Fisher-Pitman Permutation Test

data: PERC_COP by TREAT (CAF+SCTG, Full Thickness)

Z = -2.97, p-value = 0.001194

alternative hypothesis: true mu is not equal to 0

> coin::oneway_test(PERC_COP ~ TREAT, data=FullT[-c(1,8,15),], alternative="two.sided", distribution="exact")

Exact Two-Sample Fisher-Pitman Permutation Test

data: PERC_COP by TREAT (CAF+SCTG, Full Thickness)

Z = -2.4671, p-value = 0.0101

alternative hypothesis: true mu is not equal to 0

**# Cliff's delta test (test of dominance, assuming neither normality or heteroschedasticity)**

with(FullT[-c(1,8,15),], noquote(orddom(PERC_COP[TREAT=="Full Thickness"], PERC_COP[TREAT=="CAF+SCTG"], x.name="FTPGT", y.name="CAF+SCTG")))

ordinal metric

var1_X FTPGT FTPGT

var2_Y CAF+SCTG CAF+SCTG

type_title indep indep

n in X 17 17

n in Y 20 20

N #Y>X 13 13

N #Y=X 192 192

N #Y<X 135 135

PS X>Y 0.397058823529412 0.740169407826232

PS Y>X 0.0382352941176471 0.259830592173768

A X>Y 0.679411764705882 0.679411764705882

A Y>X 0.320588235294118 0.320588235294118

delta -0.358823529411765 -8.56791176470588

1-alpha 95 95

CI low -0.579151739042758 -15.2030717494928

CI high -0.0896431606093018 -1.93275177991901

s delta 0.124264362393502 9.73268535711004

var delta 0.0154416317610636 94.7251642605042

se delta <NA> 3.21065829681971

z/t score -2.88758194626627 -2.66858412593852

H1 tails p/CI 2 2

p 0.00661441822772968 0.00883554667931133

Cohen's d -0.383647416676458 -0.880323513021691

d CI low -0.570510085891556 -1.55728068848779

d CI high -0.107583177051219 -0.203366337555596

var d.i 0.0288235294117647 16.3464058823529

var dj. 0.276051720997997 160.728329210526

var dij 0.307444039562728 168.572567012841

df 35 23.4205304412417

NNT -2.78688524590164 -0.969809911032759

with(FullT[-c(1,8,15),], delta_gr(PERC_COP[TREAT=="Full Thickness"], PERC_COP[TREAT=="CAF+SCTG"], x.name="FTPGT", y.name="CAF+SCTG"))


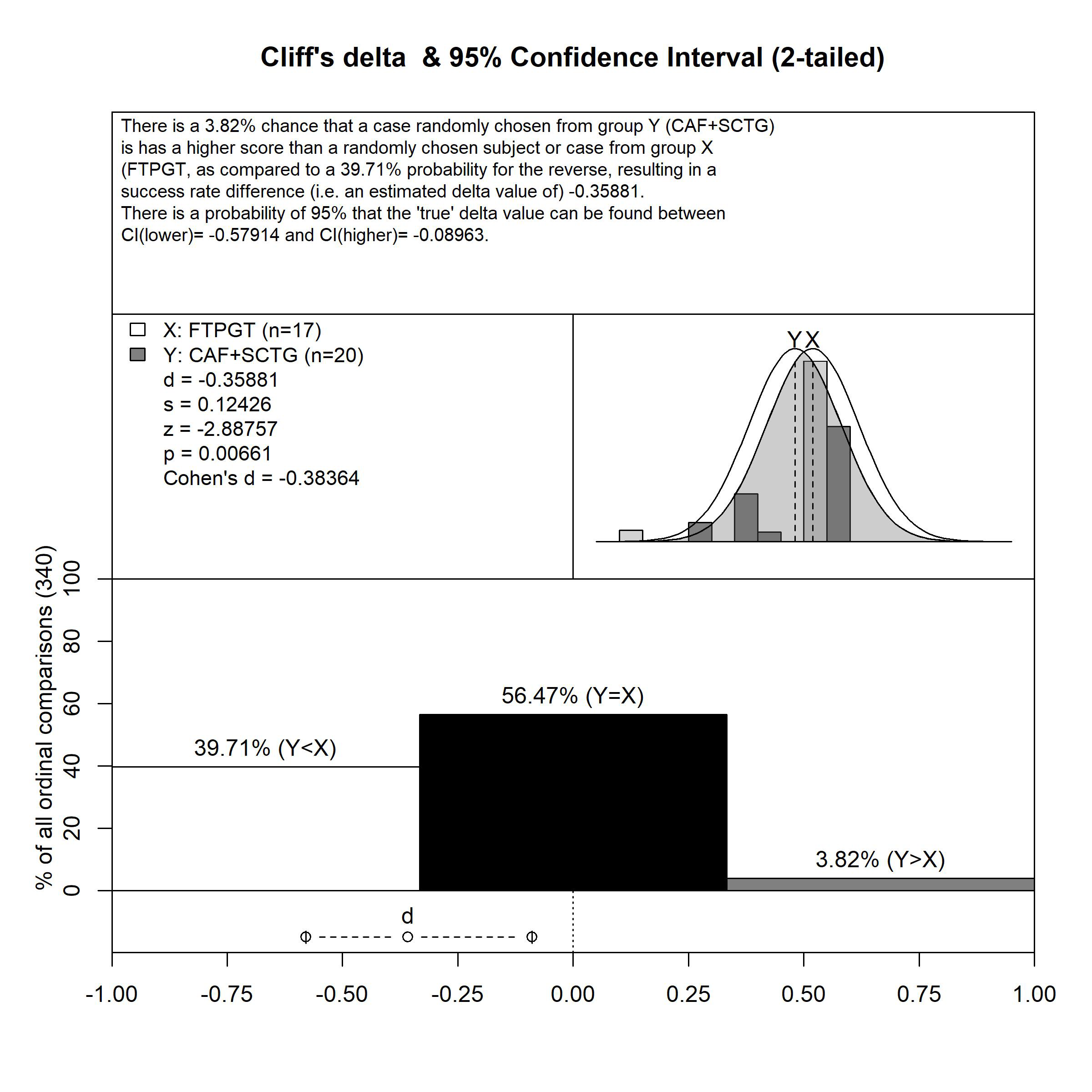


Supplementary Material 6


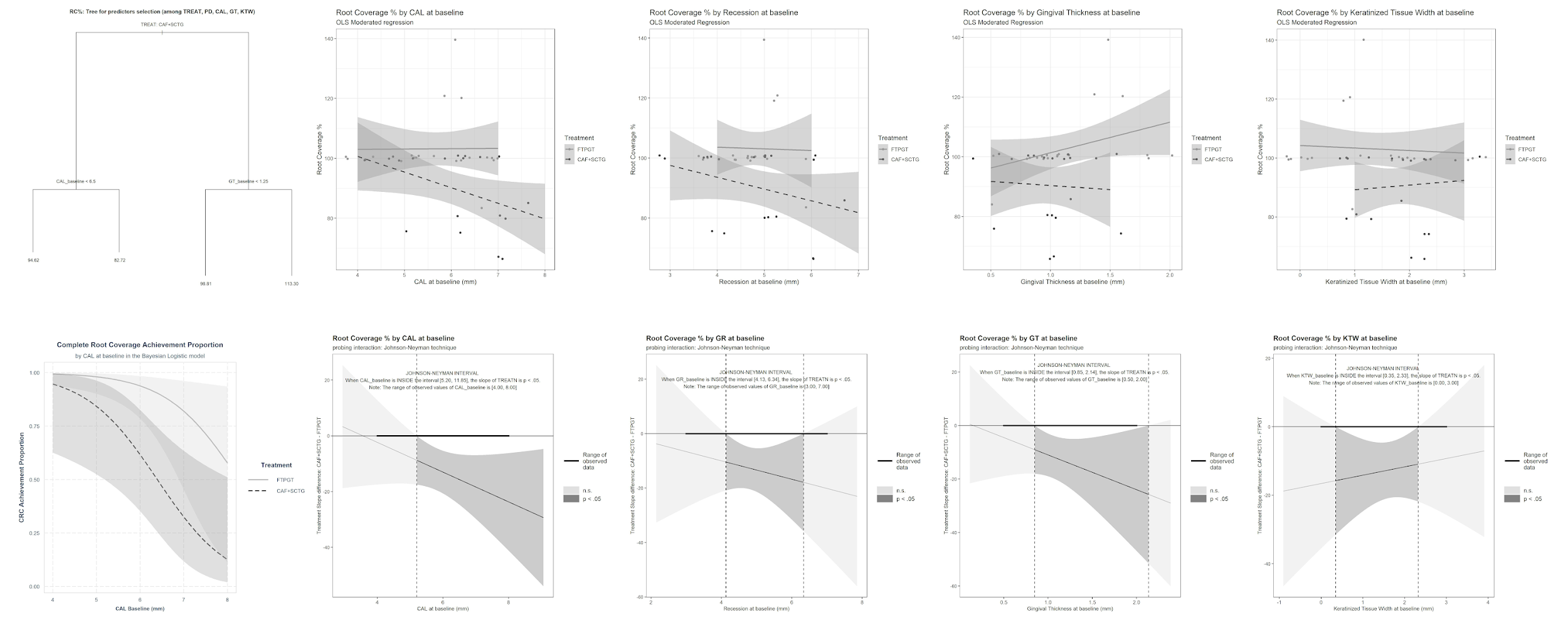


Same as Fig. 3, but with column 1 showing the more powerful model including CAL at baseline as covariate. Full sample.

First column: Tree model for predictors selection (from TREAT, PD, CAL, GT, KTW) of RC%; Bayesian Logistic Regression of the resulting model (CRC ~ CAL at baseline + TREAT) on the binary CRC outcome. Column 2-5: ordinary least squares moderated regression model on the RC% for each one of the 4 parameters – i.e. 2^nd^ column: RC% ~ CAL_baseline * TREAT with its Johnson-Neyman significance region (5.48 to 10.30).

Supplementary Material 7


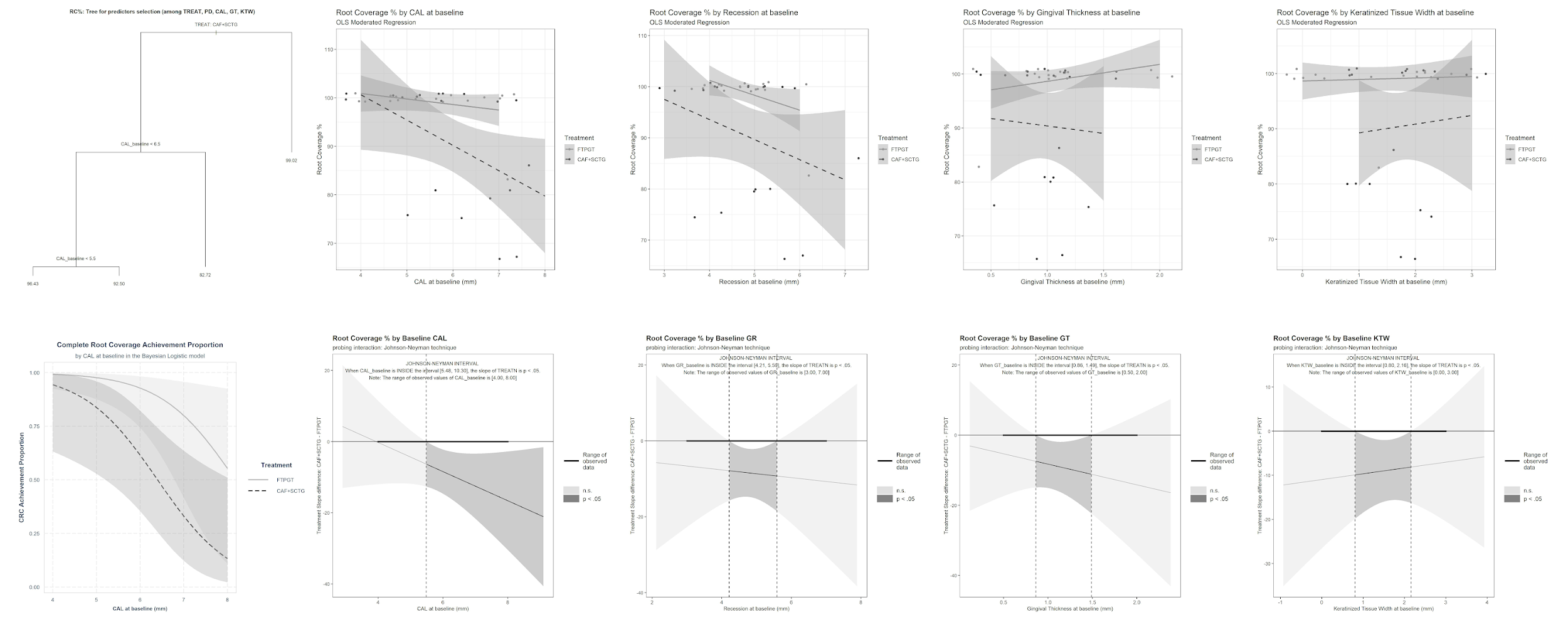


Same as SM 6, but excluding 3 RC%>100 cases.

First column: Tree model for predictors selection (from TREAT, PD, CAL, GT, KTW) of RC%; Bayesian Logistic Regression of the resulting model (CRC ~ CAL at baseline + TREAT) on the binary CRC outcome. Column 2-5: ordinary least squares moderated regression model on the RC% for each one of the 4 parameters – i.e. 2^nd^ column: RC% ~ CAL_baseline * TREAT with its Johnson-Neyman significance region (5.48 to 10.30).

Supplementary Material 8


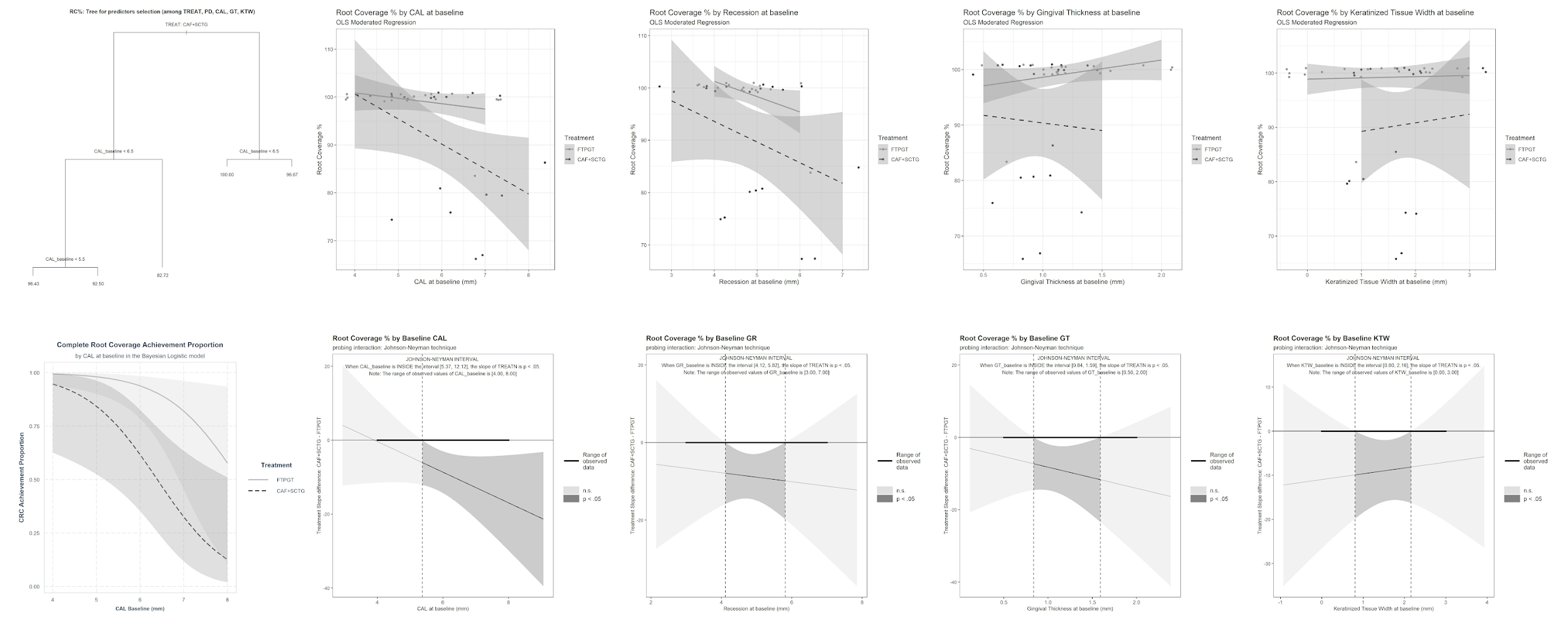


Same as SM 6, but winsorizing to 100% the 3 RC%>100 cases.

First column: Tree model for predictors selection (from TREAT, PD, CAL, GT, KTW) of RC%; Bayesian Logistic Regression of the resulting model (CRC ~ CAL at baseline + TREAT) on the binary CRC outcome. Column 2-5: ordinary least squares moderated regression model on the RC% for each one of the 4 parameters – i.e. 2^nd^ column: RC% ~ CAL_baseline * TREAT with its Johnson-Neyman significance region (5.37 to 12.12).

Supplementary Material 9

**Exploration of the treatment results according to the follow-up and gain parameters**

The Cleveland's lowess smoother in the model RC% ~ GT_T1_ provided an interesting insight about the three greater than 100% RC outliers. Specifically, the three over-covered cases (top-right) appeared outlying due to having the thickest tissue by far after the graft (5.5 mm). The graph suggests that a thickness greater than 4 mm might not be desirable.


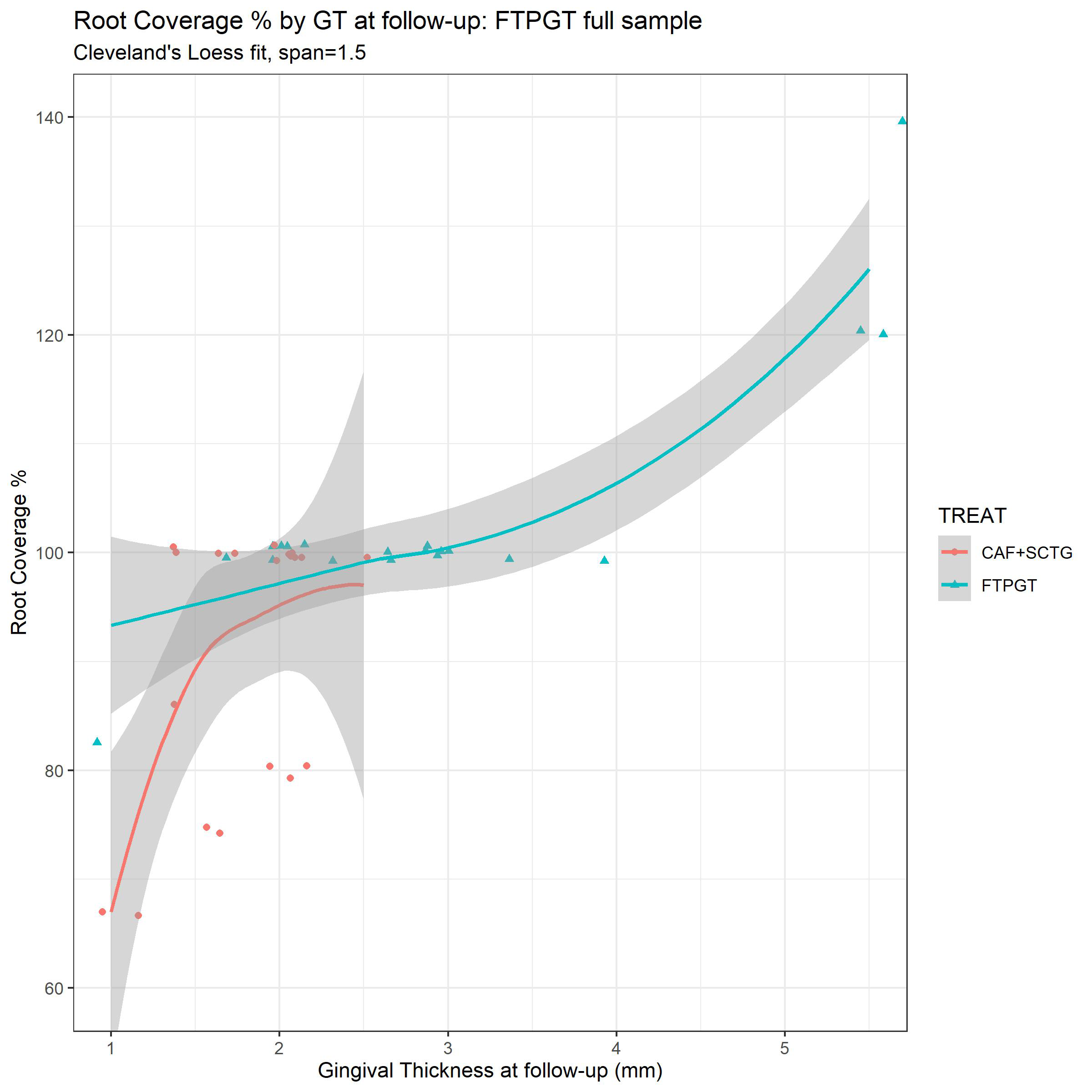


At the opposite end, the only FTPGT case (10) not achieving the CRC was also the one with the smallest thickness at follow-up (1 mm) despite gaining 0.5 mm (not the smallest increase). Either it received too small a graft, or its thickness decreased disproportionally over time—an unusually small outcome even for the CAF+SCTG treatment—or both.


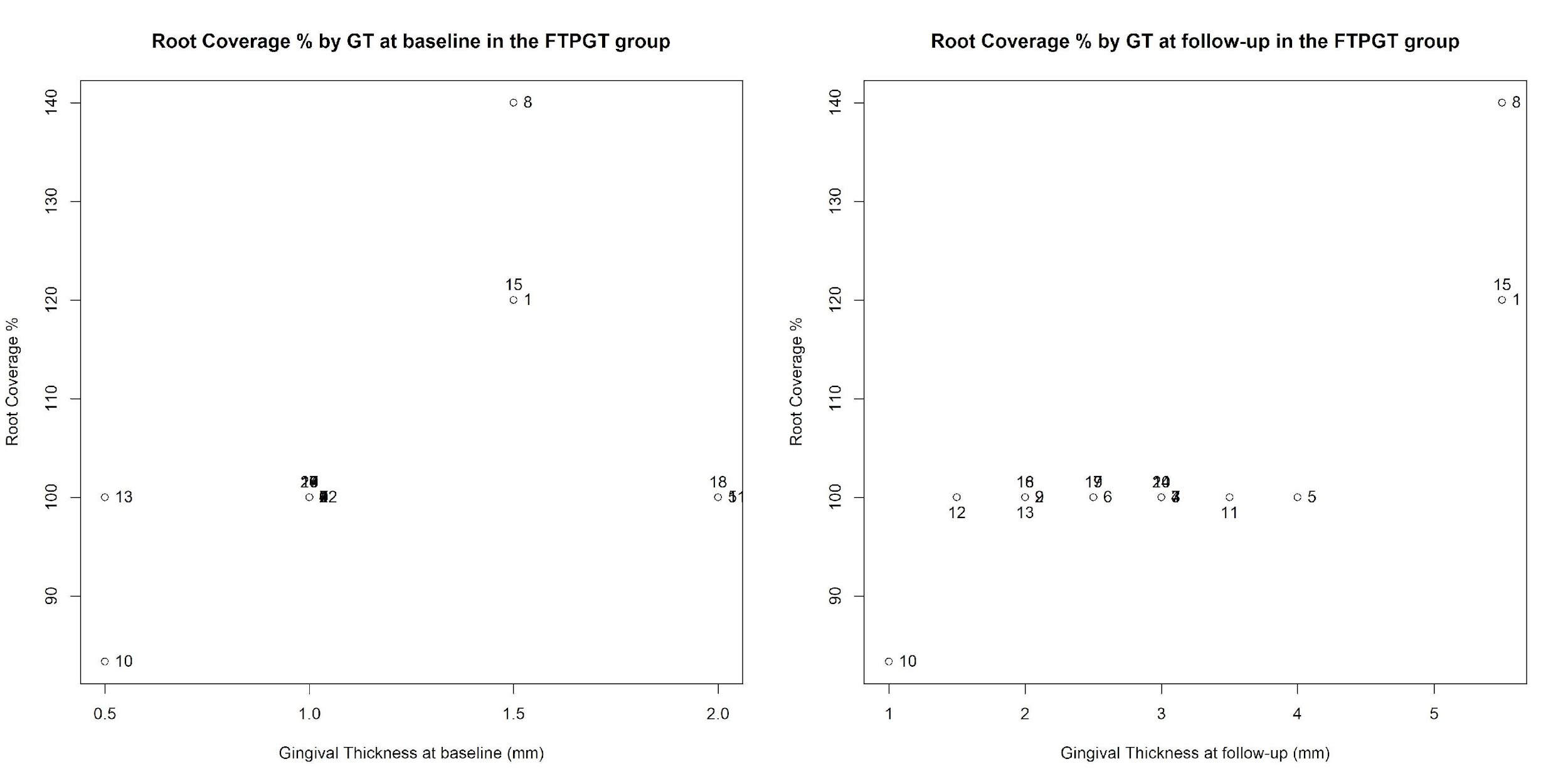


Case 10 was not alone, but among the two to have the smallest thickness at baseline (0.5 mm), as noted above. The other case (13), though, having gained 1.5 mm at follow-up, achieved CRC.

As SM 4 (at the bottom) shows, the variance of the GT change from T0 to T1 increases when the three greater than 100% RC outliers are removed, suggesting they were less susceptible to shrinkage than the average.

Supplementary Material 10

***Exploratory analysis: baseline covariates, results***

The objective of our exploration was to analyse the relationship between RC% and clinical parameters at T0. We wanted to take advantage that including the baseline measurements of the outcome as a covariate still gives rise to a design-based inference, but realised at once that something like RC% at baseline was meaningless. Therefore we wondered what parameter would be the closest, concluding that it would probably be GR at baseline. However, to validate this assumption, we opted to conduct a tree model analysis, testing the treatment variable alongside all baseline parameters. We also performed two separate analyses with reduced sets, excluding either GR or CAL due to their high collinearity (r=0.87).

**# PREDICTORS SELECTION FOR THE RC% outcome: model-based inference**

ancova.full<-lm(PERC_COP ~ TREAT + GR_baseline + CAL_baseline + GT_baseline + KTW_baseline + PD_baseline)

vif(ancova.full) **# Variance Inflation Factors: GR and CAL at baseline > 10 if both included**

TREAT GR_baseline CAL_baseline GT_baseline KTW_baseline PD_baseline

1.360092 18.287320 26.624567 1.415568 1.464254 7.341092

ancova.gr<-lm(PERC_COP ~ TREAT + GR_baseline + GT_baseline + KTW_baseline + PD_baseline)

vif(ancova.rec)

TREAT GR_baseline GT_baseline KTW_baseline PD_baseline

1.267763 1.091937 1.305832 1.463062 1.373082

ancova.cal<-lm(PERC_COP ~ TREAT + CAL_baseline + GT_baseline + KTW_baseline + PD_baseline)

vif(ancova.cal)

TREAT CAL_baseline GT_baseline KTW_baseline PD_baseline

1.276496 1.589755 1.292855 1.456037 1.765377

cor(CAL_baseline, GR_baseline) # Pearson's correlation ≈ .87

[1] 0.867859

modtree1<-tree(ancova.full);modtree2<-tree(ancova.cal);modtree3<-tree(ancova.gr)

windows(8,8);plot(modtree1);text(modtree1,pretty=0);title("Predictors selection: from TREAT, GR, CAL, GT, KTW and PD at baseline")


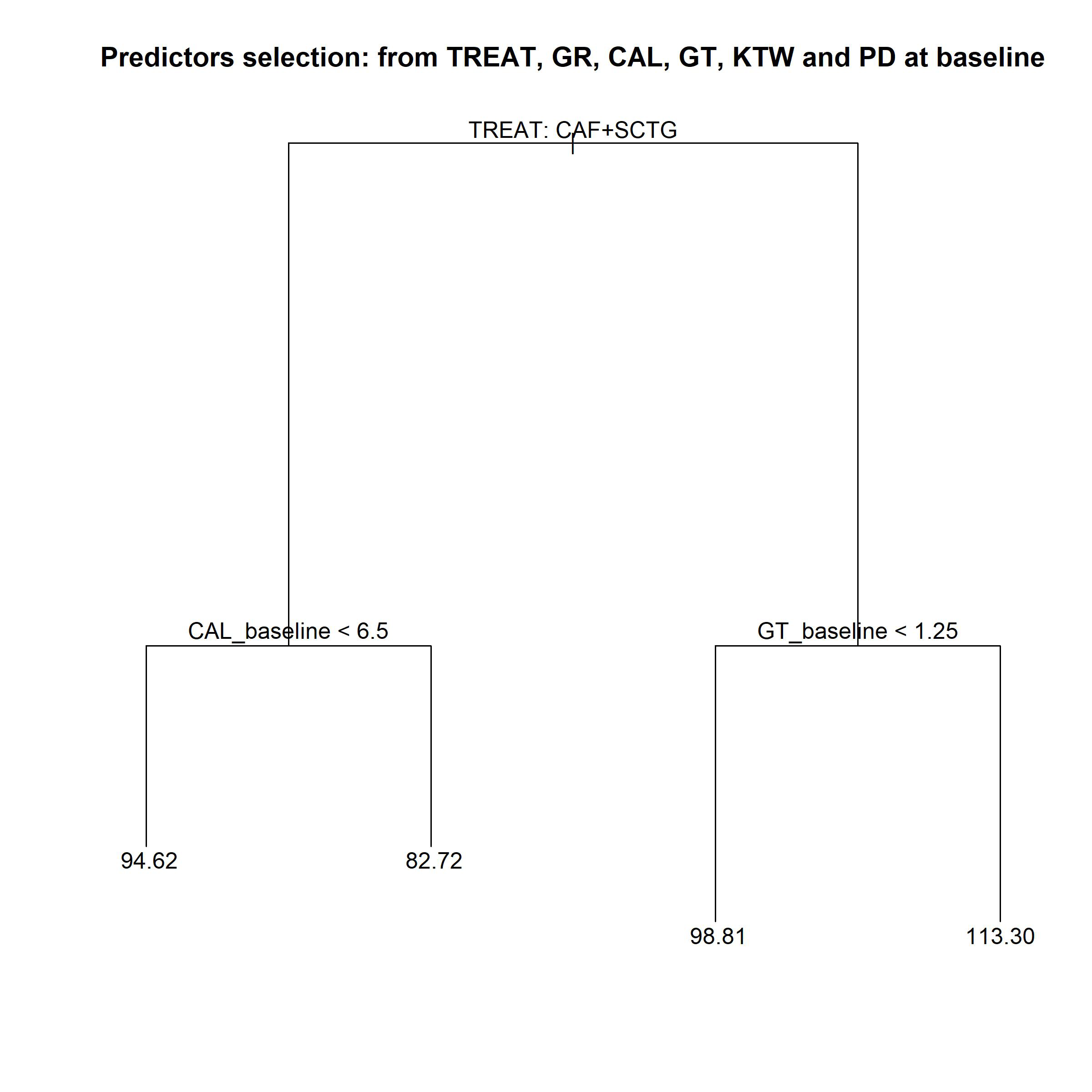


windows(16,8); par(mfrow=c(1,2)); plot(modtree2);text(modtree2, pretty=0); title("Predictors selection: from TREAT, CAL, GT, KTW and PD at baseline"); plot(modtree3);text(modtree3,pretty=0); title("Predictors selection: from TREAT, GR, GT, KTW and PD at baseline")


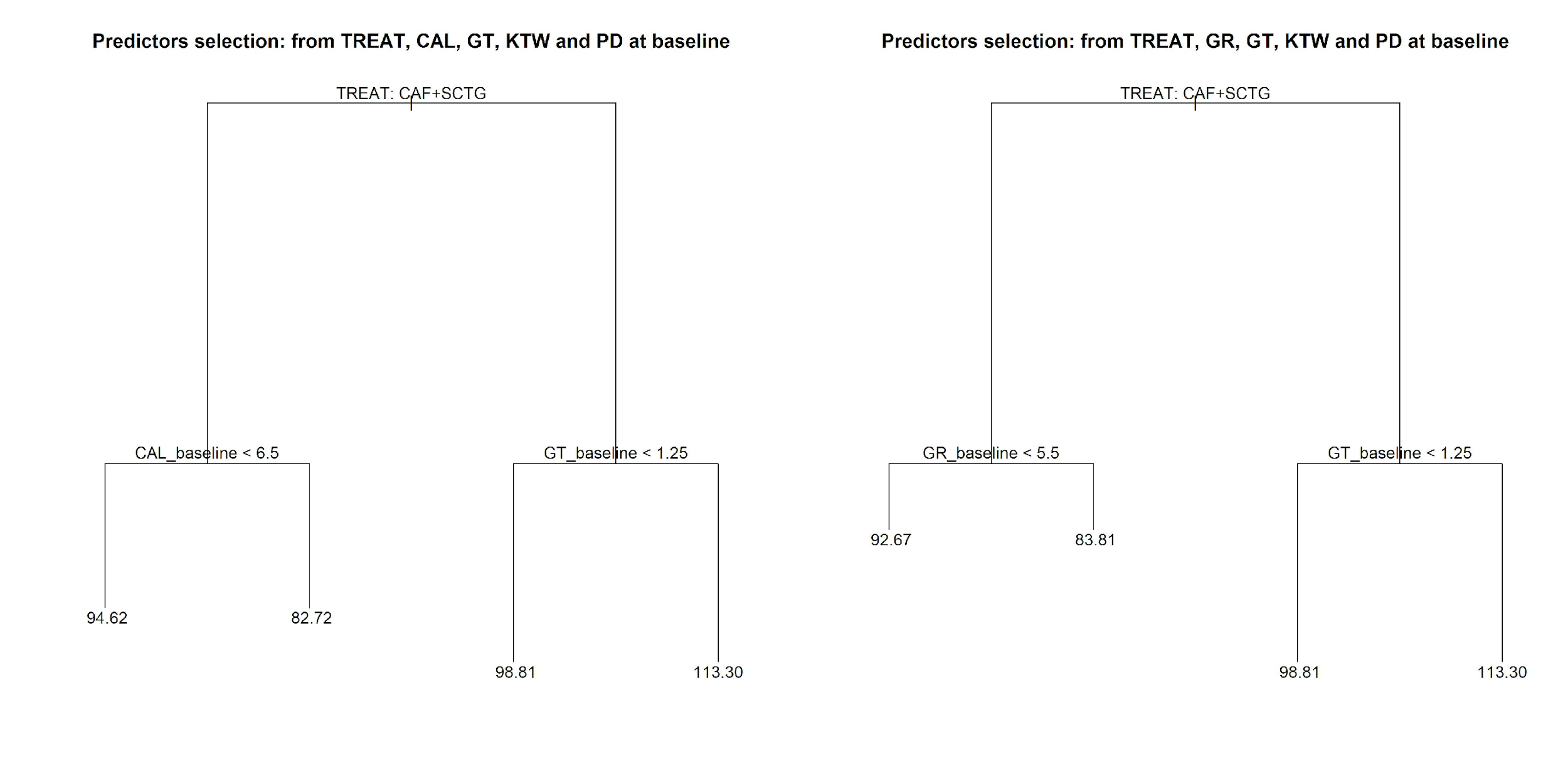


First, the selection among all the variables (upper plot) and that excluding GR (lower left) are identical. More interestingly, after the treatment predictor (tree root), the resulting next important variable among the other parameters was not the same for the two treatments. In the model at right, for CAF+SCTG, the greater the GR_T0_, the smaller the RC% (93% for GR_T0_<5.5 and 84% for GR_T0_>5.5). For FTPGT, instead, GR_T0_ explained no variance, while GT_T0_, whose worst prediction was RC≈99%, was only working to distinguish the three RC%>100 outlying cases (compare top left plots in SM 6 and 7).

These results agreed with those from the linear interactive models: the Johnson-Neyman technique showed FTPGT was superior for GR_T0_ 4.13 ÷ 6.34mm (figure below), although both the Treatment-GR_T0_ interaction (*p*=0.51) and the simple-slopes (CAF+SCTG: -3.94, *p*=0.14; FTGPT: -0.56, *p*=0.91) were inconclusive.

By replacing GR_T0_ with CAL_T0_ as covariate, however, FTPGT (simple-slope=0.12, *p*=0.95) resulted superior to CAF+SCTG (simple-slope=-5.22, *p*=0.02) for *every* CAL_T0_>5.2mm, being the interaction term still inconclusive (*p*=0.14)^^[[1]](#footnote-1)^^.


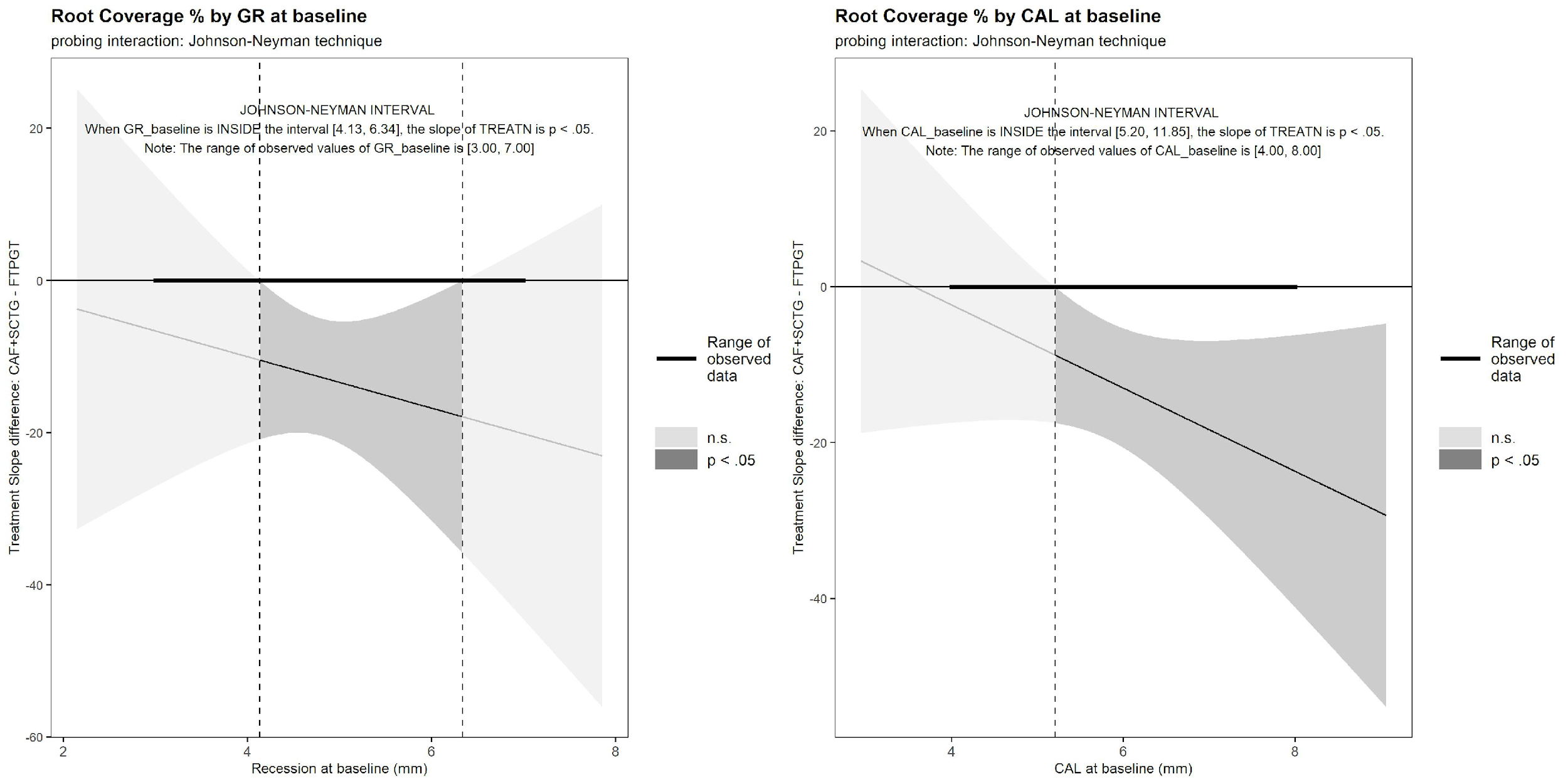


interactions::sim_slopes(COP.int.rec, pred=GR_baseline, modx=TREAT, robust=T)

SIMPLE SLOPES ANALYSIS

Slope of GR_baseline when TREAT = Full Thickness:

Est. S.E. t val. p

------- ------ -------- ------

-0.56 4.64 -0.12 0.91

Slope of GR_baseline when TREAT = CAF+SCTG:

Est. S.E. t val. p

------- ------ -------- ------

-3.94 2.58 -1.53 0.14

Messaggio di avvertimento:

Johnson-Neyman intervals are not available for factor moderators.

interactions::sim_slopes(COP.int.cal, pred=CAL_baseline, modx=TREAT, robust=T)

SIMPLE SLOPES ANALYSIS

Slope of CAL_baseline when TREAT = Full Thickness:

Est. S.E. t val. p

------ ------ -------- ------

0.12 1.81 0.06 0.95

Slope of CAL_baseline when TREAT = CAF+SCTG:

Est. S.E. t val. p

------- ------ -------- ------

-5.22 2.13 -2.45 0.02

Messaggio di avvertimento:

Johnson-Neyman intervals are not available for factor moderators.

The power analysis of the model including CAL_T0_ (power 33%) indicates that, with an *α*=.05, 125 patients would be needed to get 80% power for the interaction term. The model including GR_T0_ (power 22.9%) would require instead 190 patients to get the same power.


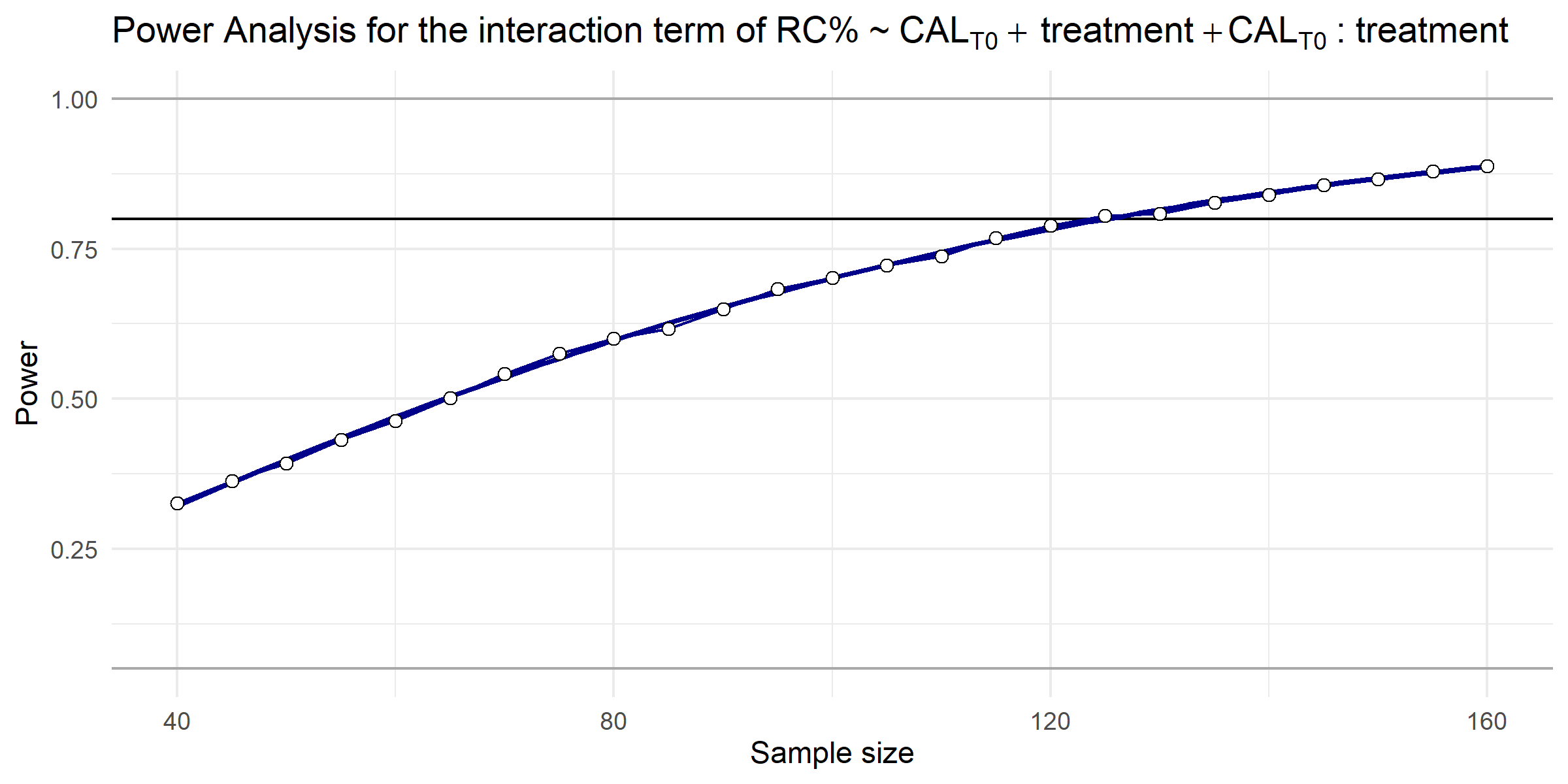


Monte Carlo simulations show the FTPGT slope estimates include zero for sample size at least until 220. The CAF+SCTG slope, conversely, is consistently negative (CAF+SCTG IQR in yellow, FTPGT in black), supporting the FTPGT flatness against CAF+SCTG negative slope.

**
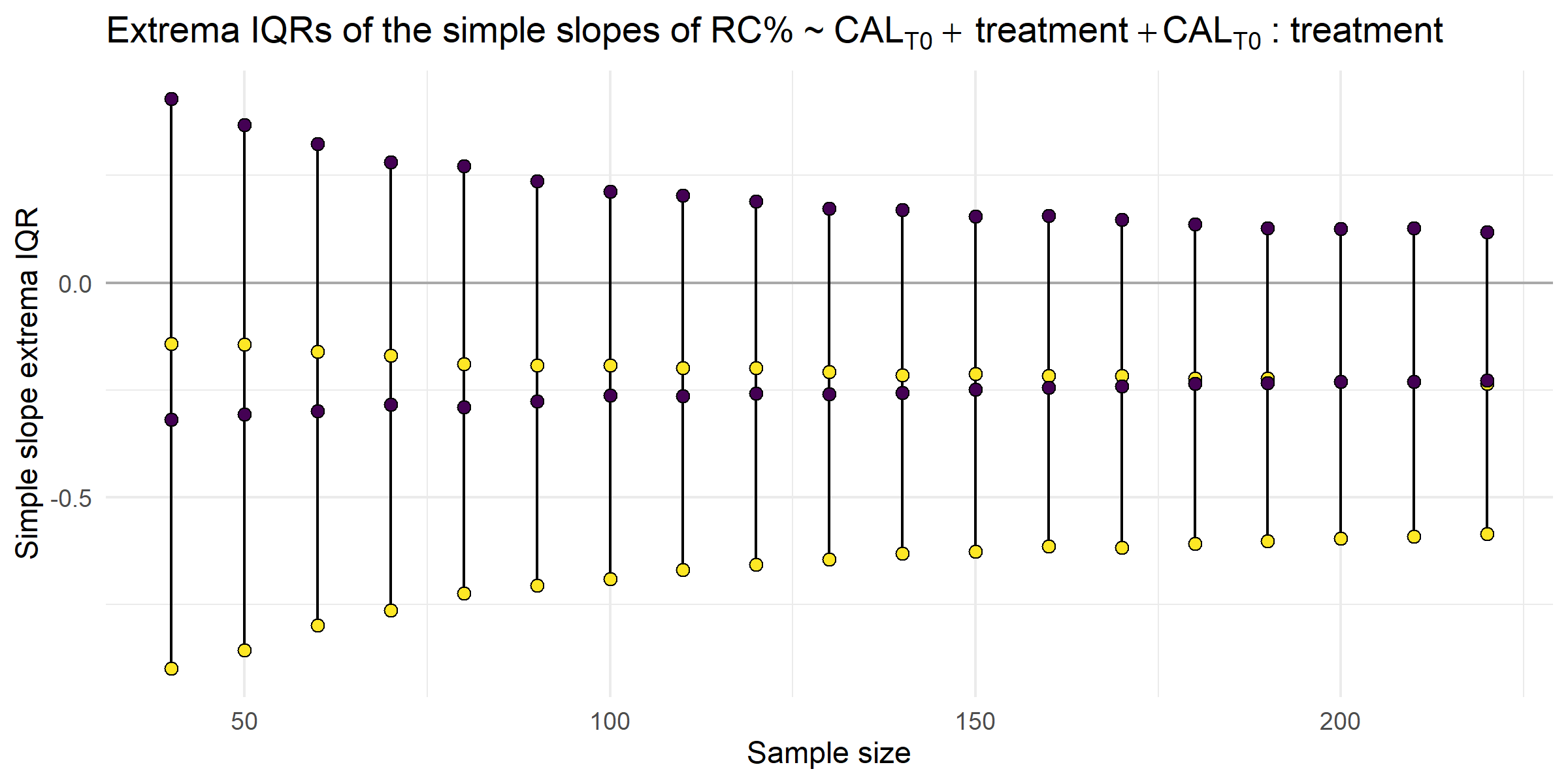
**

**# INTERACTIVE MODELS COMPARISON: GR vs CAL**

COP.int.cal<-lm(PERC_COP ~ TREAT * CAL_baseline); summary(COP.int.cal)

Call:

lm(formula = PERC_COP ~ TREAT * CAL_baseline)

Residuals:

Min 1Q Median 3Q Max

-20.411 -3.316 -3.086 4.932 36.799

Coefficients:

Estimate Std. Error t value Pr(>|t|)

(Intercept) 121.513 14.731 8.249 0.000000000815 ***

TREATFTPGT -19.004 21.047 -0.903 0.373

CAL_baseline -5.220 2.437 -2.142 0.039 *

TREATFTPGT:CAL_baseline 5.336 3.561 1.498 0.143

---

Signif. codes: 0 ‘***’ 0.001 ‘**’ 0.01 ‘*’ 0.05 ‘.’ 0.1 ‘ ’ 1

Residual standard error: 11.67 on 36 degrees of freedom

Multiple R-squared: 0.3137, Adjusted R-squared: 0.2565

F-statistic: 5.486 on 3 and 36 DF, p-value: 0.003292

COP.int.gr<-lm(PERC_COP ~ TREAT * GR_baseline); summary(COP.int.gr)

Call:

lm(formula = PERC_COP ~ TREAT * GR_baseline)

Residuals:

Min 1Q Median 3Q Max

-19.169 -3.611 -3.055 6.399 36.945

Coefficients:

Estimate Std. Error t value Pr(>|t|)

(Intercept) 109.347 12.815 8.533 0.00000000036 ***

TREATFTPGT -3.511 25.173 -0.139 0.890

GR_baseline -3.937 2.610 -1.508 0.140

TREATFTPGT:GR_baseline 3.380 5.184 0.652 0.519

---

Signif. codes: 0 ‘***’ 0.001 ‘**’ 0.01 ‘*’ 0.05 ‘.’ 0.1 ‘ ’ 1

Residual standard error: 12.02 on 36 degrees of freedom

Multiple R-squared: 0.2725, Adjusted R-squared: 0.2118

F-statistic: 4.494 on 3 and 36 DF, p-value: 0.008872

nonnest2::vuongtest(COP.int.cal, COP.int.gr)

Model 1

Class: lm

Call: lm(formula = PERC_COP ~ TREAT * CAL_baseline)

Model 2

Class: lm

Call: lm(formula = PERC_COP ~ TREAT * GR_baseline)

Variance test

H0: Model 1 and Model 2 are indistinguishable

H1: Model 1 and Model 2 are distinguishable

w2 = 0.016, p = 0.0817

Non-nested likelihood ratio test

H0: Model fits are equal for the focal population

H1A: Model 1 fits better than Model 2

z = 1.481, p = 0.0693

H1B: Model 2 fits better than Model 1

z = 1.481, p = 0.9307

AIC(COP.int.rec, COP.int.cal)

df AIC

COP.int.rec 5 318.2199

COP.int.cal 5 315.8842

lmtest::lrtest(COP.anc.cal, COP.int.cal)

Likelihood ratio test

Model 1: PERC_COP ~ TREAT + CAL_baseline

Model 2: PERC_COP ~ TREAT * CAL_baseline

#Df LogLik Df Chisq Pr(>Chisq)

1 4 -154.15

2 5 -152.94 1 2.4194 0.1198

BIC(COP.int.rec, COP.int.cal)

df BIC

COP.int.rec 5 326.6643

COP.int.cal 5 324.3286

lmtest::lrtest(COP.anc.rec, COP.int.rec)

Likelihood ratio test

Model 1: PERC_COP ~ TREAT + GR_baseline

Model 2: PERC_COP ~ TREAT * GR_baseline

#Df LogLik Df Chisq Pr(>Chisq)

1 4 -154.34

2 5 -154.11 1 0.4696 0.4932

For CRC, the same outcome (RC) but dichotomized, the Cochran-Mantel-Haenszel test provides similar results whether including CAL_T0_ or GR _T0_ as stratification variables, confirming they convey pretty much the same information.

**#### Cochran-Mantel-Haenszel test: CAL stratifying variable, FULL SAMPLE**

**# Collapsing last stratum to avoid sparse data**

FullT$CAL<-ifelse(CAL_baseline<7,CAL_baseline,7);FullT$CAL

[1] 6 7 7 5 4 6 6 6 7 7 5 4 5 5 6 7 5 4 6 6 6 4 5 4 7 5 5 6 7 5 5 7 6 6 7 7 6 6 7 7

tabella2<-table(COP_SN,TREAT,CAL);tabella2 # COP_SN->CRC yes/no,

, , CAL = 4

TREAT

COP_SN CAF+SCTG Full Thickness

NO 0 0

SI 2 3

, , CAL = 5

TREAT

COP_SN CAF+SCTG Full Thickness

NO 1 0

SI 4 5

, , CAL = 6

TREAT

COP_SN CAF+SCTG Full Thickness

NO 2 0

SI 4 7

, , CAL = 7

TREAT

COP_SN CAF+SCTG Full Thickness

NO 5 1

SI 2 4

mantelhaen.test(tabella2, correct=FALSE)

Mantel-Haenszel chi-squared test without continuity correction

data: tabella2

Mantel-Haenszel X-squared = 6.3071, df = 1, p-value = 0.01203

alternative hypothesis: true common odds ratio is not equal to 1

95 percent confidence interval:

1.351874 280.167819

sample estimates:

common odds ratio

19.46154

mantelhaen.test(tabella2, exact=TRUE)

Exact conditional test of independence in 2 x 2 x k tables

data: tabella2

S = 8, p-value = 0.01544

alternative hypothesis: true common odds ratio is not equal to 1

95 percent confidence interval:

1.201672 735.158729

sample estimates:

common odds ratio

12.91543

DescTools::WoolfTest(tabella2)

Woolf Test on Homogeneity of Odds Ratios (no 3-Way assoc.)

data: tabella2

X-squared = 3.0208, df = 3, p-value = 0.3884

**# GR stratifying variable**

**# Collapsing last stratum to avoid sparse data**

FullT$REC<-ifelse(GR_baseline<6,GR_baseline,6);detach(FullT);attach(FullT)

tabella4<-table(COP_SN,TREAT,REC);tabella4

, , REC = 3

TREAT

COP_SN CAF+SCTG FTPGT

NO 0 0

SI 2 0

, , REC = 4

TREAT

COP_SN CAF+SCTG FTPGT

NO 2 0

SI 4 6

, , REC = 5

TREAT

COP_SN CAF+SCTG FTPGT

NO 3 0

SI 4 12

, , REC = 6

TREAT

COP_SN CAF+SCTG FTPGT

NO 3 1

SI 2 1

mantelhaen.test(tabella4, correct=F)

Mantel-Haenszel chi-squared test without continuity correction

data: tabella4

Mantel-Haenszel X-squared = 6.221, df = 1, p-value = 0.01262

alternative hypothesis: true common odds ratio is not equal to 1

95 percent confidence interval:

1.118732 120.934829

sample estimates:

common odds ratio

11.63158

mantelhaen.test(tabella4, exact=T)

Exact conditional test of independence in 2 x 2 x k tables

data: tabella4

S = 8, p-value = 0.03186

alternative hypothesis: true common odds ratio is not equal to 1

95 percent confidence interval:

1.133715 534.033056

sample estimates:

common odds ratio

10.72982

DescTools::WoolfTest(tabella4)

Woolf Test on Homogeneity of Odds Ratios (no 3-Way assoc.)

data: tabella4

X-squared = 3.0208, df = 3, p-value = 0.3884

Regarding GT_T0_, once again, both the simple-slope were inconclusive (FTPGT=10.24, *p*=0.19; CAF+SCTG=-2.75, *p*=0.8). The significance region indicated FTPGT's superiority from 0.85 to 2.14.

A similar scenario unfolded with KTW. The KTW_T0_ by treatment interactive model yielded coefficients >0.05, while their simple slopes were CAF+SCTG=1.58, p=0.70 and FTPGT=0.86, p=0.42. The significance region indicated CAF+SCTGis inferiority to FTPGT for KTW_T0_ values between 0.35 and 2.33 mm, *p*=0.05 (as illustrated in Fig. 3). However, the results for KTW_T0_<0.35 mm results seemed questionable, possibly due to insufficient power.


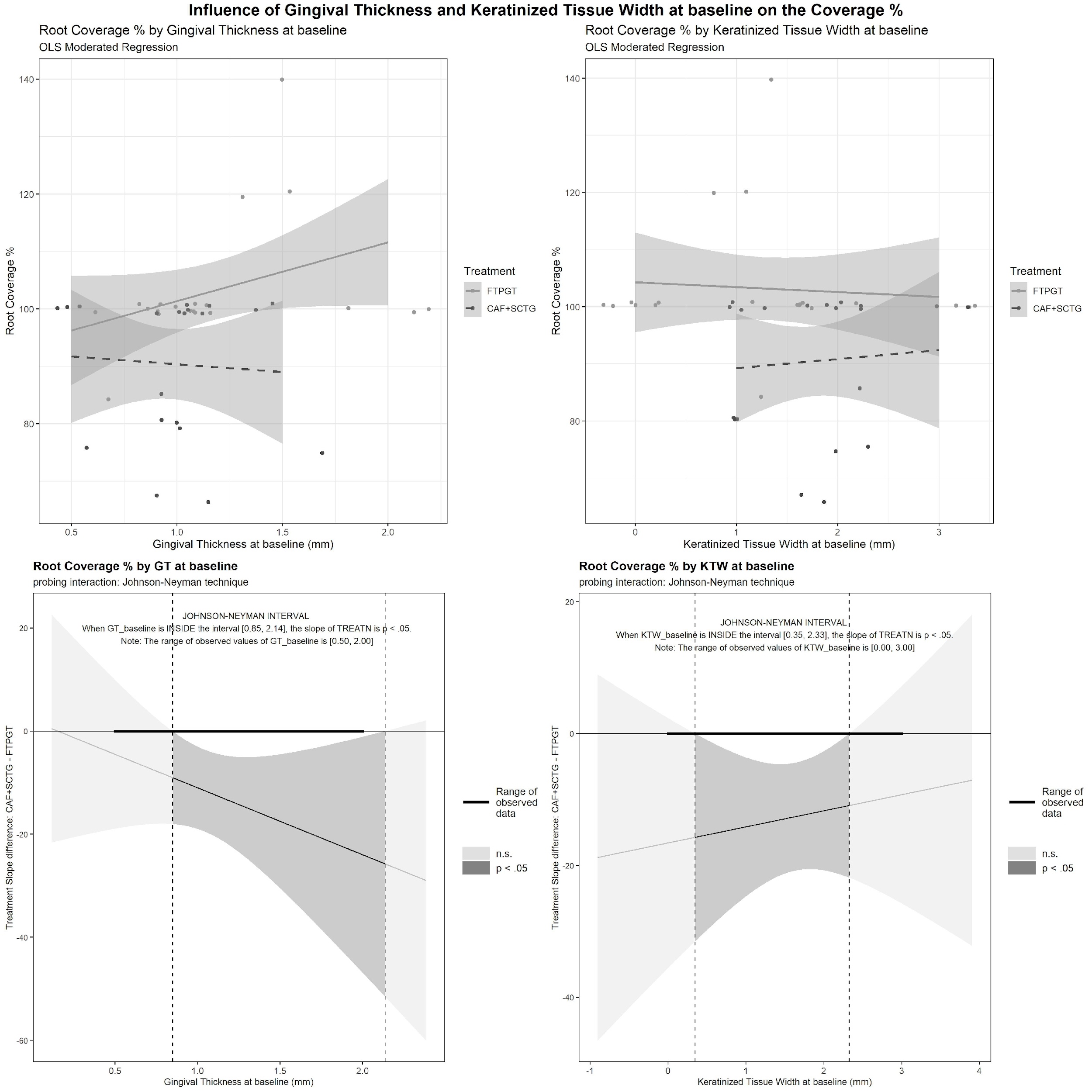


Supplementary material 11

***Baseline covariates: discussion***

Is leaving a "non-significant" covariate in a model incorrect practice? Several authors disagree (Kullback–Leibler information against NHST)^^[[2]](#footnote-2)^^. The Akaike weights are^^[[3]](#footnote-3)^^:

myout<-lmtest::lrtest(COP.anc.cal, COP.int.cal)

myout$deltaAIC<-myout$Chisq-2*myout$Df

myout$w.r<-exp(-.5*myout$deltaAIC) / (1 + exp(-.5*myout$deltaAIC))

myout$w.f<-1-myout$w.r

myout[, -c(3:4)]

#Df LogLik Pr(>Chisq) deltaAIC w.r w.f

1 4 -154.15

2 5 -152.94 0.11984 0.41945 0.44776 0.55224

therefore CAL_T0_ should be included (its *p*<0.15) according to the Senn's (1994) *tomorrow's standard*^^[[4]](#footnote-4)^^, while all sensible covariates should according to the *after tomorrow's* one.

Both models including GR_T0_ (W_F_=.32) or CAL_T0_ (W_F_=.55) as covariate show a similar behaviour, leading to one prediction: CAF+SCTG loses efficacy for GR_T0_>4 mm (or CAL_T0_>5) while FTPGT does not. The CAL_T0_ simple slopes and their power analysis appear to agree.

For GT_T0_ and KTW_T0_, since their values are radically modified by the interventions, the question arises why they should still continue to have an effect at the follow-up. The answer is that these measures provide prognostic information because the tissue present at baseline is responsible for blood supply to the graft.

The sole portion of the CAF+SCTG graft not receiving blood supply is that facing the root, all the others being in contact with vascularized tissues, the flap or the recipient bed, and therefore the graft receives good supply even from a very thin flap, less rich in vessels. In the FTPGT, instead, a portion of the graft remains exposed, so a smaller surface has to guarantee the blood supply, resulting more dependent on the flap characteristics, its thickness and abundance in the content of vessels. It should be considered, however, that the detected FTPGT dependency on GT_T0_ vanishes once excluding the three outliers, while its worst expectation remains RC≈99%, dominating CAF+SCTG for all GT_T0_>0.85.

From our data, the magnitude of KTW_T0_ does not influence the RC% of FTPGT because this technique involves covering the exposed root entirely with keratinized tissue, regardless of baseline KTW. On the contrary, the keratinization induced by the graft in the CAF+SCTG technique is of reduced extent (just over 1 mm^7^) and does not occur immediately. This observation may suggest to the clinician the use of FTPGT in RT1s belonging to Miller class II.

The treatment mechanics makes the GT_T1_ and KTW_T1_ measurements replace those at T0, so eventually they explain from a new perspective the treatments' efficacy. For instance, the literature reports that CAF+SCTG may increase KTW by approximately 1 mm^^[[5]](#footnote-5)^^; here we showed FTPGT is able to increase KTW by an average of 4.2 mm^^[[6]](#footnote-6)^^.


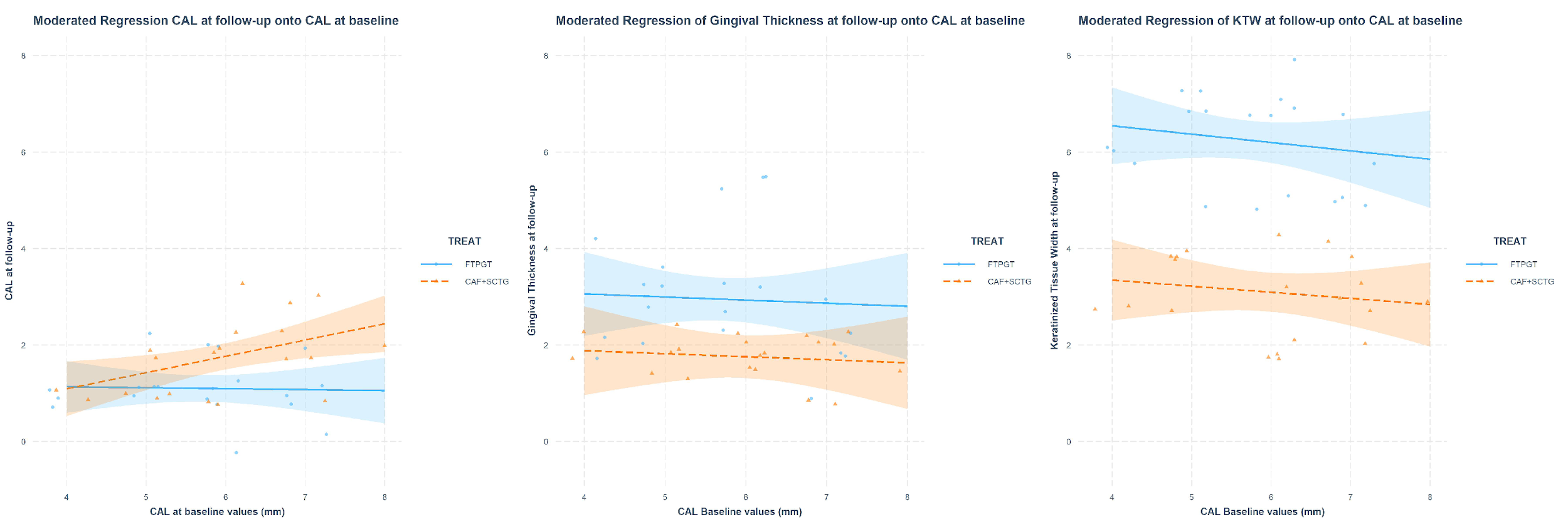


Supplementary material 12

**SENSITIVITY ANALYSIS: Cox's model robustness to proportional hazard assumption**


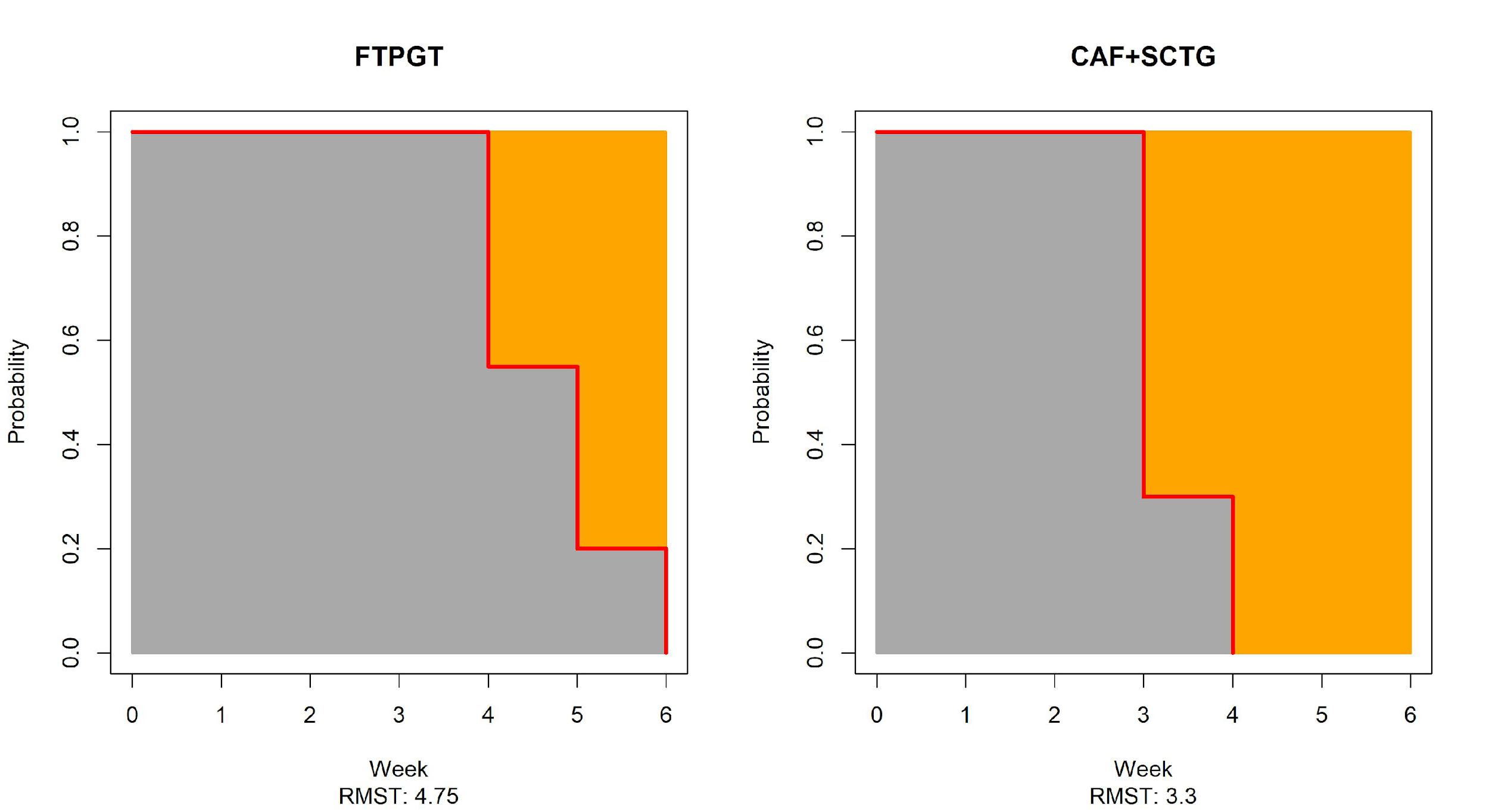


Restricted Means Survival Time Analysis of the Healing times as a sensitivity analysis of Cox's model

library(survRM2);rmst<-with(FTHeal, rmst2(H.W, STATUS, arm=as.numeric(TREAT)-1));rmst

The truncation time, tau, was not specified. Thus, the default tau 6 is used.

Restricted Mean Survival Time (RMST) by arm

Est. se lower .95 upper .95

RMST (arm=1) 4.75 0.171 4.414 5.086

RMST (arm=0) 3.30 0.102 3.099 3.501

Restricted Mean Time Lost (RMTL) by arm

Est. se lower .95 upper .95

RMTL (arm=1) 1.25 0.171 0.914 1.586

RMTL (arm=0) 2.70 0.102 2.499 2.901

Between-group contrast

Est. lower .95 upper .95 p

RMST (arm=1)-(arm=0) 1.450 1.059 1.841 0

RMST (arm=1)/(arm=0) 1.439 1.311 1.580 0

RMTL (arm=1)/(arm=0) 0.463 0.350 0.612 0

> rmst$unadjusted.result

Est. lower .95 upper .95 p

RMST (arm=1)-(arm=0) 1.450000 1.0586202 1.8413798 0.00000000000038323240

RMST (arm=1)/(arm=0) 1.439394 1.3111706 1.5801566 0.00000000000001992561

RMTL (arm=1)/(arm=0) 0.462963 0.3503056 0.6118506 0.00000006195614484658

Supplementary Material 13

**
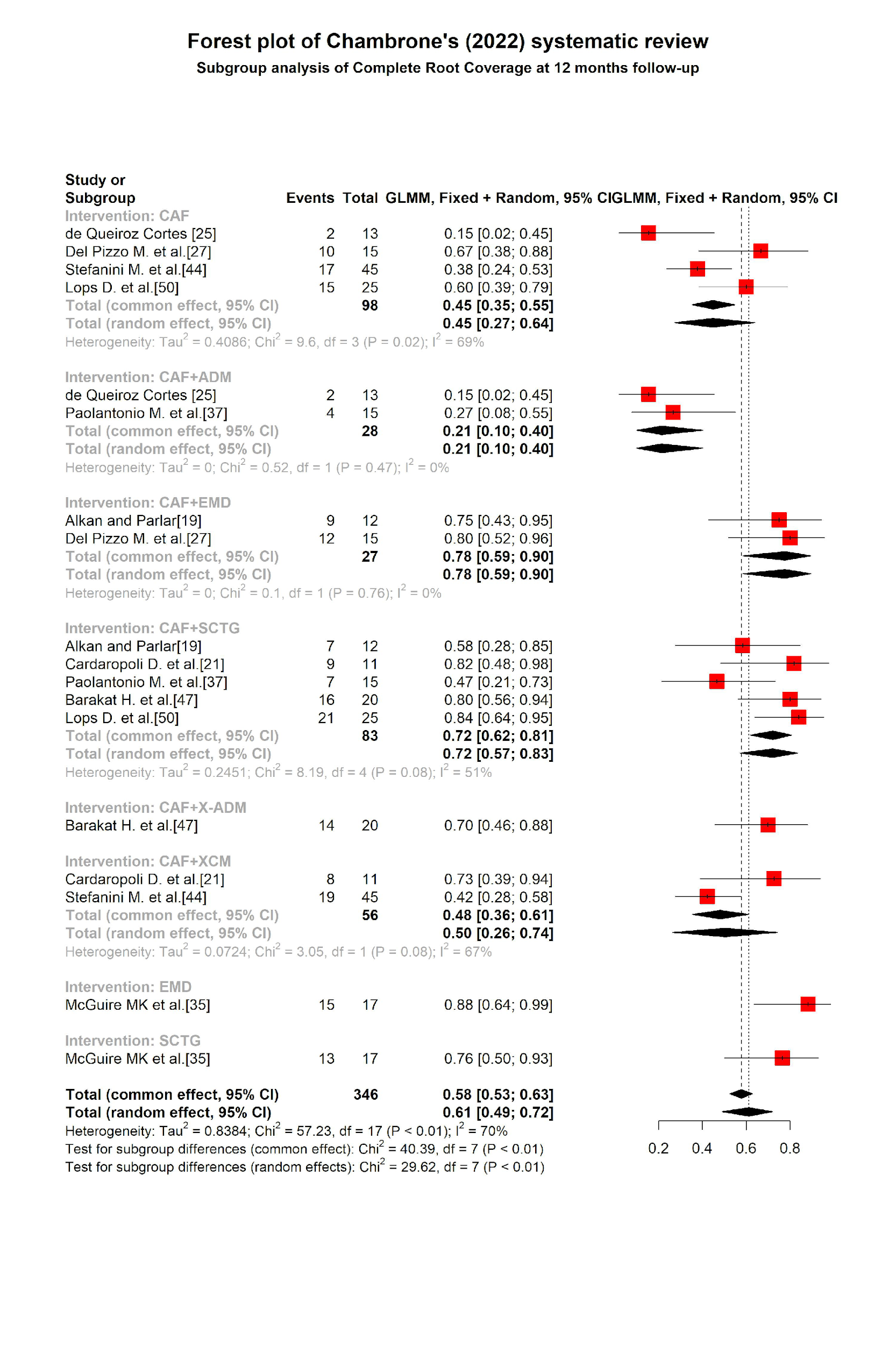
**

Meta-analysis of Chambrone's (2022) systematic review studies on CAF+SCTG reporting CRC at a 12 months follow-up, with subgroup analysis.

1. Hayes AF. Introduction to mediation, moderation, and conditional process analysis: a regression-based approach. Third edition. New York London: The Guilford Press; 2022. [↑](#footnote-ref-1)
2. Rothman, K. J., Greenland, S., & Lash, T. L. (2008). Modern epidemiology (Vol. 3). Philadelphia: Wolters Kluwer Health/Lippincott Williams & Wilkins [↑](#footnote-ref-2)
3. Long, J. D. (2012). *Longitudinal data analysis for the behavioral sciences using R*. Sage., ¶ 8.3 [↑](#footnote-ref-3)
4. Senn, S. (1994). Testing for baseline balance in clinical trials. Statistics in medicine, 13(17), 1715-1726. [↑](#footnote-ref-4)
5. Cairo F, Nieri M, Pagliaro U. Efficacy of periodontal plastic surgery procedures in the treatment of localised facial gingival recessions. A systematic review. J Clin Periodontol [Internet]. 2014 Apr [cited 2023 Nov 19];41(s15). Available from: <https://onlinelibrary.wiley.com/doi/10.1111/jcpe.12182> [↑](#footnote-ref-5)
6. Paolantonio M, De Ninis P, Secondi L, Femminella B. The Full-Thickness Palatal Graft Technique: Description of an Original Surgical Technique and 15 Case Reports. Int J Periodontics Restorative Dent. 2019 Feb;39(2):267–77. [↑](#footnote-ref-6)
